# Subcellular chemical mapping using correlated cryogenic electron and mass spectrometry imaging

**DOI:** 10.1038/s41592-026-03109-7

**Published:** 2026-05-25

**Authors:** Hannah Ochner, Buse Isbilir, Sonja Blasche, David Scheidweiler, Yuexuan Zhang, Zhexin Wang, Tom Smith, Catarina Franco, Rob Bradley, Kiran R. Patil, Tanmay A. M. Bharat

**Affiliations:** 1 Structural Studies Division, MRC Laboratory of Molecular Biology, Cambridge, UK; 2 MRC Toxicology Unit, University of Cambridge, Cambridge, UK; 3 Cell Biology Division, MRC Laboratory of Molecular Biology, Cambridge, UK

**Keywords:** Molecular imaging, Cryoelectron microscopy, Mass spectrometry, Cellular microbiology

## Abstract

Electron cryomicroscopy (cryo-EM) allows high spatial resolution visualization of biological specimens; however, it is challenging to chemically identify densities observed in cryo-EM. To overcome this, we combined cryo-EM with chemical imaging using focused ion beam secondary ion mass spectrometry (FIB-SIMS) for integrated spatiochemical analysis of untagged specimens. We show that our correlative workflow permits subcellular localization of molecules inside bacterial cells and is compatible with cryogenic light microscopy and FIB-milled lamellae of eukaryotic specimens. To highlight biological insights enabled by the workflow, we studied the uptake of bisphenol-AF, a widespread chemical pollutant, by environmental bacteria, revealing the storage of these chemicals within cytosolic phase-separated aggregates in pollutant-exposed cells, where they cannot be removed by the bacterial efflux machinery despite its robust upregulation. Cryo-EM-FIB-SIMS therefore represents an effective approach to map elemental and molecular signatures in near-native biological samples.

## Abstract (web summary)

A correlative workflow combining cryo-EM and focused ion beam secondary ion mass spectrometry (FIB-SIMS) facilitates subcellular chemical analysis with high spatial resolution.

## Main

Electron cryomicroscopy (cryo-EM) provides high spatial resolution for visualizing a wide range of biological samples from purified molecules to cells and tissues^1–3^; however, there is no direct way of assigning chemical identity to the densities observed in electron micrographs, which means that different molecules present within the sample cannot be easily distinguished, especially within the cellular context^1^. Mass spectrometry (MS), on the other hand, is a chemically specific technique, allowing direct chemical identification of the molecules in a sample, thus providing highly complementary information to cryo-EM structural data. While many MS techniques are performed on bulk samples, MS can also be conducted in an imaging modality, which allows simultaneous detection of the spatial distributions of different molecules in a specimen^4–8^. In particular, secondary ion mass spectrometry (SIMS), utilizing a range of different ion beams for the pixel-by-pixel ionization of the specimen, in conjunction with extraction of the resulting secondary ions into mass analyzers such as time-of-flight (ToF-SIMS), Orbitrap (OrbiSIMS) and magnetic sector mass spectrometers, has been shown to be a versatile tool for studying the chemical composition of biological specimens^9–17^. While SIMS applications range from single cell to tissue imaging, and from elemental ion detection to metabolomics and proteomics^9–14,18^, the attainable spatial resolution is limited by the characteristics of the primary ion beam^6^. To detect molecules of interest (or their fragments) along with their subcellular localization in untagged specimens, single-cell measurements with both high spatial resolution and chemical sensitivity are required. Examples of biological processes that would benefit from such imaging include, but are not limited to, drug uptake by cells, pollutant uptake by bacteria and symbiotic metabolite sharing within microbiomes. Thus, combining the capabilities of cryo-EM and imaging MS would extend the capabilities of both these techniques, providing images with unprecedented explanatory power for mechanistic structural and cellular studies of a wide range of biological processes.

To this end, we have developed a workflow to perform correlated cryo-EM with MS imaging of vitrified biological specimens using a focused ion beam (FIB)-SIMS instrument at cryogenic temperatures. We provide proof-of-principle data for tagged and untagged biological specimens and extend our approach to show that the technique is compatible with cryo-light microscopy (cryo-LM), as well as with thicker specimens, such as eukaryotic cells, which can be imaged in the form of FIB-milled lamellae. These examples demonstrate the versatility of the spatiochemical imaging workflow, which can be tailored to the requirements of the biological specimen. To highlight the utility of our integrated workflow, we studied bioaccumulation of environmental contaminants in bacterial cells. Specifically, we studied the uptake of a widespread fluorinated pollutant, bisphenol-AF (BPAF), by environmental bacteria, showing that these molecules are concentrated in the cytosol inside phase-separated aggregates and cannot be removed by the drug efflux machinery despite its upregulation by the bacteria. Our results on BPAF bioaccumulation underscore the complementarity of cryo-EM and MS imaging, showing how effectively combining the two imaging modalities supports biological discovery.

## Results

### Integrated cryo-EM and FIB-SIMS imaging workflow

**Fig. 1:**
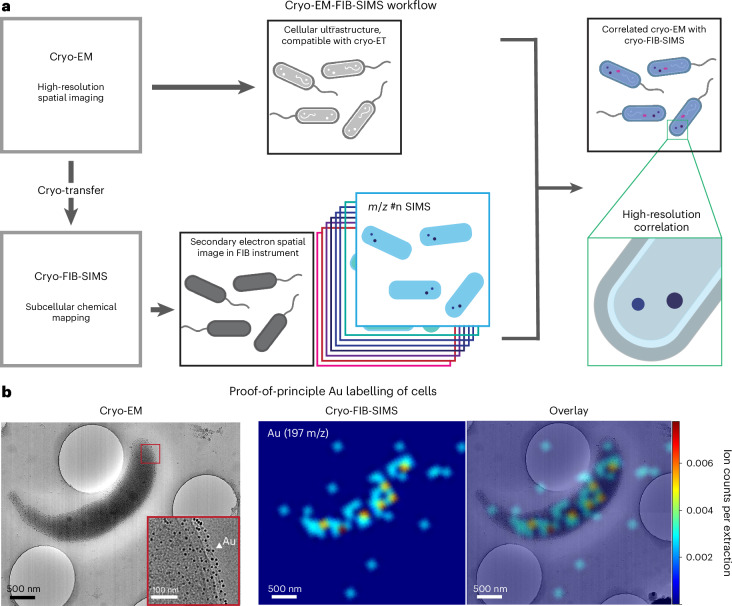
Correlative cryo-EM-FIB-SIMS workflow. **a**, Schematic of the cryo-EM-FIB-SIMS workflow. As a first step, vitrified specimens are imaged by cryo-EM, yielding high-resolution spatial images detailing the cellular ultrastructure. Subsequently, the samples are transferred to the cryo-FIB-SIMS instrument where imaging MS is performed on the sample regions pre-imaged by cryo-EM. In addition to the chemical information from SIMS imaging, secondary electron spatial FIB images are also collected which are useful for correlation of the cryo-FIB-SIMS and the cryo-EM data. The data from both imaging modalities can then be overlaid, connecting the chemical information from cryo-FIB-SIMS with the high-resolution spatial information from cryo-EM. **b**, Proof-of-principle example of the workflow using gold nanoparticle-labeled *C.* *crescentus* cells. Left: the S-layer of the cells has been labeled with gold nanoparticles resulting in a coating of the entire cell with gold as demonstrated by cryo-EM (gold nanoparticles appear as black dots around the cell). The inset is a high-magnification image of the area highlighted by the red box; gold nanoparticles are indicated by a white arrowhead. Right: this is confirmed by the cryo-FIB-SIMS data which show that the gold (Au, 197 *m*/*z*) signal colocalizes with the cell and is distributed along its length. The color scale of the MS images encodes the detected ion count per extraction. Panel **a** created in BioRender; Wang, Z. https://biorender.com/pr5u156 (2026).

**Extended Data Fig. 1:**
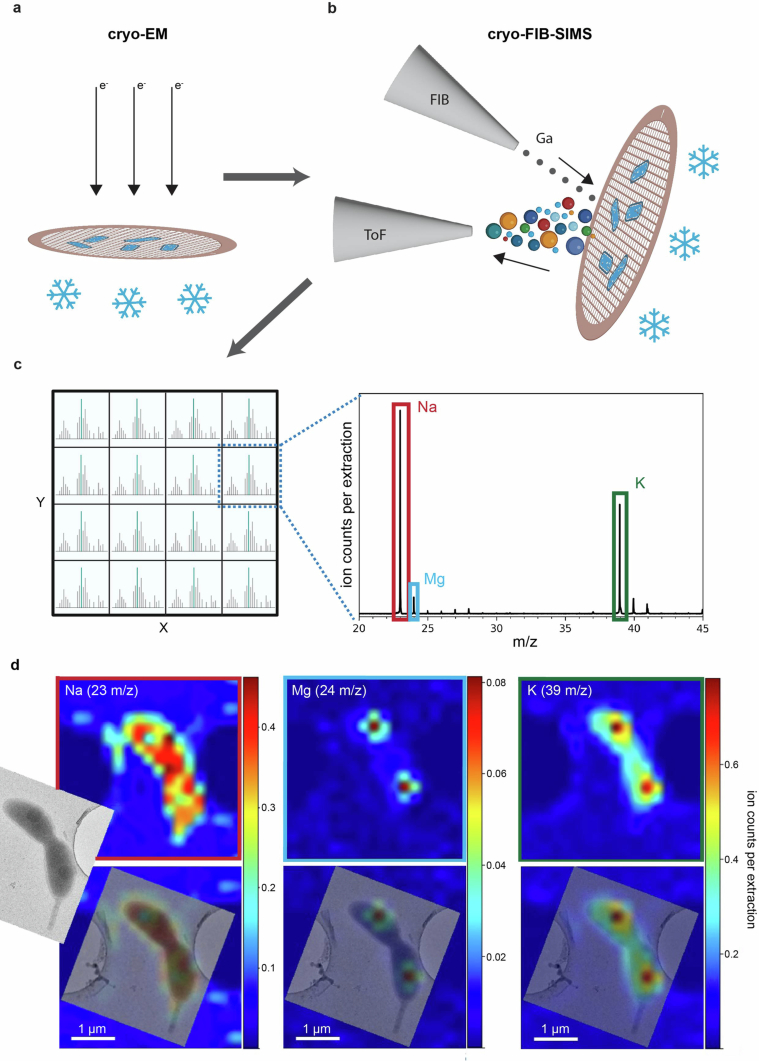
Cryo-EM-FIB-SIMS workflow. **a**-**d** Cryo-EM-FIB-SIMS working principle: after cryo-EM imaging (**a**), the same specimen area is imaged by cryo-FIB-SIMS. The primary gallium FIB scans the sample, ablating material during specimen interaction (**b**). At each pixel, the resulting secondary ions are extracted into the ToF mass spectrometer, yielding a mass spectrum at every pixel (**c**). From this, a 2D matrix of mass spectra, that is the spatial distribution of each detected mass-to-charge ratio (*m/z*) peak can be extracted, as exemplified for a *C. crescentus* cell in (**d**). Positive ion mode correlative analysis of a *C. crescentus* cell showing the cryo-FIB-SIMS data of three prominent *m/z* peaks (Na, Mg, and K, top) as well as overlays with the cryo-EM image (bottom). The color scale of the MS images corresponds to the detected ion count per extraction. Panel **a** created in BioRender; Wang, Z. https://biorender.com/r24d171 (2026).

**Extended Data Fig. 2:**
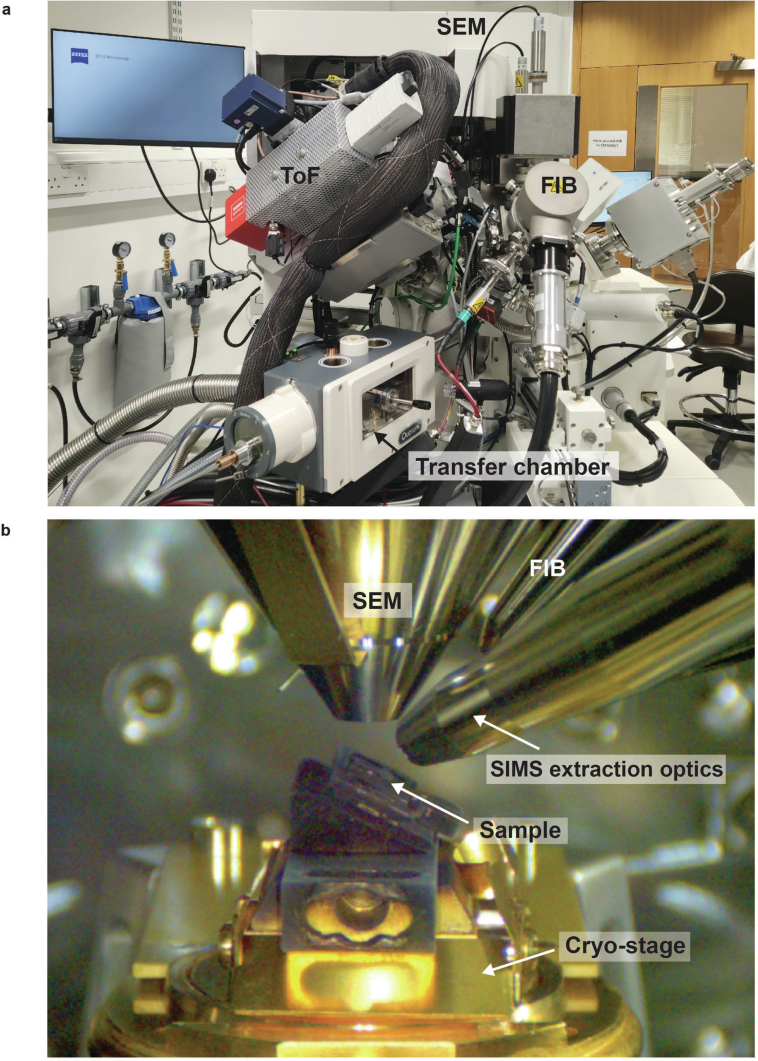
Cryo-FIB-SIMS instrument setup. **a** Photograph of the outside of the machine with relevant components marked. **b** Photograph of the inside of the microscope chamber with SIMS extraction optics inserted. Relevant components have been highlighted.

**Extended Data Fig. 3:**
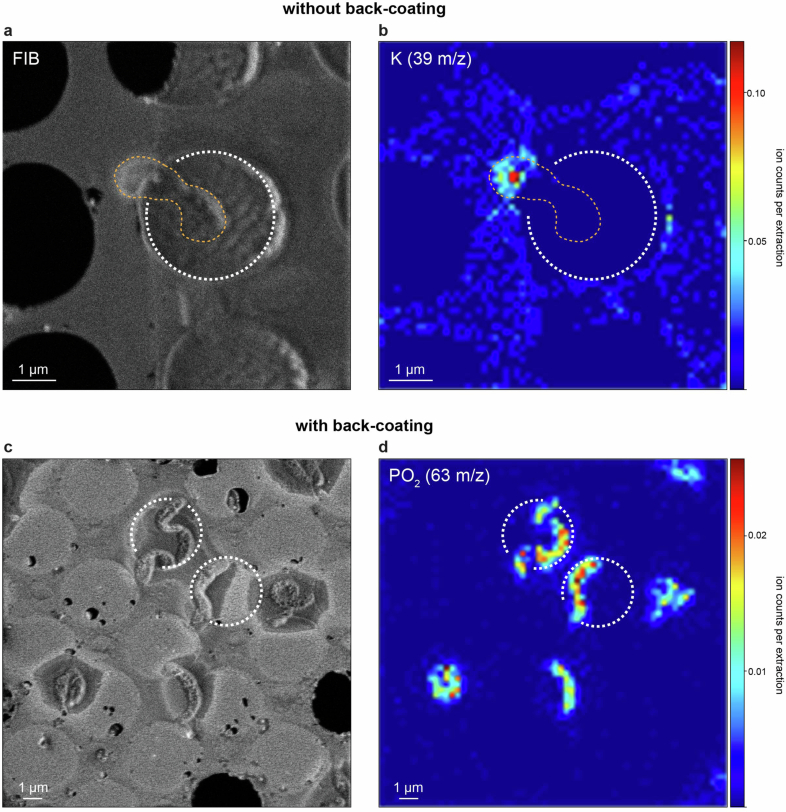
Increase of sample stability and MS signal from holey supports by back-coating. **a**-**b** FIB image (**a**) and cryo-FIB-SIMS distribution of potassium (**b**) in a *C. crescentus* cell on a holey amorphous carbon support without back-coating. In the FIB image, warping of the holes on the left can be observed. Cryo-FIB-SIMS signal is observed from the part of the cell on the carbon support, but not from the cellular area located within the hole in the support foil. **c**-**d** FIB image (**c**) and cryo-FIB-SIMS distribution of PO_2_ (**d**) of *C. crescentus* cells on holey amorphous carbon support with back-coating. The additional stability due to the organometallic coating reduces warping and facilitates the detection of the cryo-FIB-SIMS signal from the holey areas of the support film.

We developed a correlative workflow enabling chemical mapping at the subcellular scale in bacteria (Fig. 1a) under cryogenic conditions in vitrified specimens (Methods). First, two-dimensional (2D) cryo-EM images of the sample are collected (Extended Data Fig. 1a), followed by specimen transfer to the cryo-FIB-SIMS instrument (Extended Data Fig. 2) in which MS imaging is performed on the areas pre-imaged by cryo-EM to allow correlation of the cryo-EM and cryo-FIB-SIMS datasets. During specimen transfer, the stability of the sample is enhanced by applying an organometallic coating to the backside of the sample, leading to an improved MS signal as sample movement and inhomogeneities of the sample support are reduced (Extended Data Fig. 3). The cryo-FIB-SIMS instrument is a focused ion beam scanning electron microscope (FIB-SEM) equipped with a time-of-flight (ToF) mass spectrometer. To perform SIMS imaging, a focused gallium (Ga) ion beam oriented perpendicular to the sample plane (Extended Data Fig. 2b), with an unbinned pixel size range in MS imaging mode of approximately 10–30 nm, is used to scan the field of view, ablating material from the specimen (Extended Data Fig. 1b). The secondary ions produced by this ablation are extracted into the ToF mass spectrometer (at nearly the same angular orientation as the FIB) at every pixel of the scan, yielding a mass spectrum for each pixel.

**Extended Data Fig. 4:**
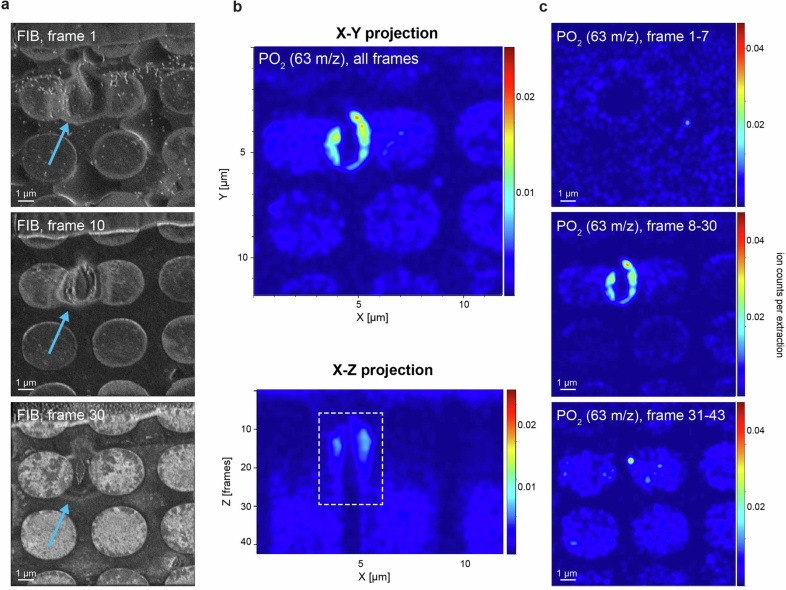
Probing the 3D chemical composition of biological specimens with cryo-FIB-SIMS. **a** Secondary electron (FIB) images of the sample corresponding to different frames, that is different heights along the ‘Z’-stack. At frame 1 (top), the cell (marked by blue arrow) is still covered in ice. By frame 10 (center), the beam is probing the interior of the cell. At frame 30 (bottom), the cellular material has been fully ablated by the beam. Thus, repeatedly scanning over the area of interest provides 3D information about the sample. **b** Corresponding cryo-FIB-SIMS image of the same cell as in **a** for 63 *m/z* (PO_2_) in an X-Y projection of all frames in the stack (top) and an X-Z slice through the Z-stack (bottom). The thickness of the volume shown here (43 frames in total) can be estimated to be between 500-700 nm based on known sputter rates of vitrified ice for Ga beams and the mean thickness of *C. crescentus* cells. While the X-Y projection shows the lateral distribution of the PO_2_ signal, the X-Z slice yields depth information and allows the localization of the cell along the Z-axis, where cellular signal can be detected from frames 8-30 (white dashed box). This is confirmed by X-Y images resulting from projections of specific frames as shown in **c**: the X-Y projection of frames 1–7 (top) does not show cellular signal and corresponds to the ice layer enveloping the cell. The X-Y projection of frames 8-30 shows a cellular image, in accordance with the cellular localization seen in the FIB images and the X-Z slice (**a**, **b**). After frame 30 (bottom, frame 31-43), the cell is not visible any more as at that point, it has been fully removed by the interaction with the ion beam (see also Supplementary Video S1).

The resulting data are a 2D matrix of mass spectra, allowing the extraction of the spatial distribution of ion counts for each detected mass-to-charge ratio (*m/z*) peak (Extended Data Fig. 1c,d). The mass spectrometer can be operated in positive or negative ion mode, which corresponds to voltage settings for extracting either positive or negative secondary ions, respectively. As material is continuously removed by the FIB, scanning the region of interest multiple times enables the recording of three-dimensional (3D) information of the chemical composition of the sample as every 2D image corresponds to a slice orthogonal to the FIB direction, cutting through the sample volume (Extended Data Fig. 4 and Supplementary Video 1). The data collected from cryo-EM and cryo-FIB-SIMS for each region of interest can then be correlatively analyzed to reveal insights into the chemical composition of the sample overlaid with ultrastructural features revealed by cryo-EM (Fig. 1a and Extended Data Fig. 1d).

**Extended Data Fig. 5:**
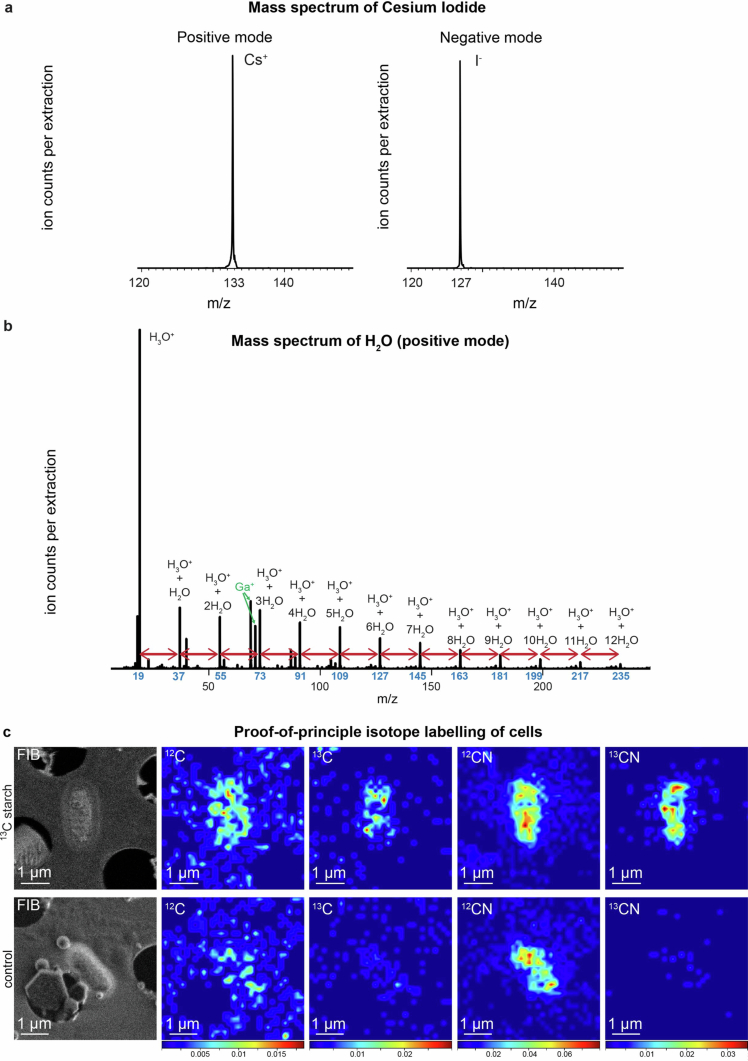
Mass spectra of vitrified standard chemicals and isotope labelling. **a** Main peaks in positive and negative ion mode mass spectra of a vitrified sample of CsI: 133 *m/z*, corresponding to Cs^+^, and 127 *m/z*, corresponding to I^−^ are detected. **b** Positive mode mass spectrum of a vitrified sample of H_2_O showing characteristic water clusters: the main peak is 19 *m/z* (H_3_O^+^) followed by peaks spaced 18 *m/z* apart, corresponding to clusters with a linearly increasing number of H_2_O molecules. Peaks due to gallium are highlighted in green. **c** Cryo-FIB-SIMS imaging of *Bacteroides thetaioataomicron* in media supplemented with ^13^C-labeled starch (top, number of cells analyzed: n = 22, two biological replicates) and a control culture grown in unlabeled media with glucose as the carbon source (bottom, number of cells analyzed: n = 13, two biological replicates). On the left, secondary electron FIB images are provided to clarify the spatial localization of the cells. Cells exposed to the ^13^C-labeled starch show clear intracellular signal of both ^13^C and ^13^CN, while the cells in the control culture do not exhibit ^13^C-related SIMS signals.

Initial testing of the FIB-SIMS system on standard chemical samples, such as a vitrified cesium iodide solution (CsI; Extended Data Fig. 5a) and vitrified water (Extended Data Fig. 5b) showed all expected ionic peaks. Next, we showed the applicability of the technique to vitrified biological specimens by imaging *Caulobacter* *crescentus* cells which were coated with gold nanoparticles by labeling of the surface layer (S-layer) protein RsaA^19,20^. The gold nanoparticles are clearly visible in cryo-EM imaging, appearing as black dots around the cell, which indicates successful coating of the entire cell surface (Fig. 1b). Imaging the same cells using cryo-FIB-SIMS reveals a clear gold signal (Au, 197 *m/z*) localized on the cells (Fig. 1b), confirming that our correlative workflow can reveal structural as well as chemical details about specifically labeled specimens. Discrepancies between the cryo-FIB-SIMS gold signal and the distribution of gold nanoparticles in the cryo-EM image (in Fig. 1b) are likely the result of the difference in resolution between the two imaging modalities as well as nonuniform ionization yields across the cell. As SIMS can distinguish elemental isotopes^21,22^, our workflow is also compatible with more subtle labels. To illustrate this, we incubated the gut bacterium *Bacteroides* *thetaiotaomicron* in a medium containing ^13^C-labeled starch and subsequently imaged the resulting sample by cryo-FIB-SIMS (Extended Data Fig. 5c). The data shows strong cellular signals corresponding to both ^13^C and ^13^CN, with the presence of ^13^CN suggesting that the starch had been metabolized to organic molecules containing both C and N. As expected, cells from the control culture did not exhibit these signals. These examples indicate how heavy metal or isotope labeling can aid the application of cryo-FIB-SIMS to biological questions related to protein localization or the uptake of chemicals into cells.

### Correlative spatiochemical imaging of bacterial cells at subcellular resolution

**Fig. 2:**
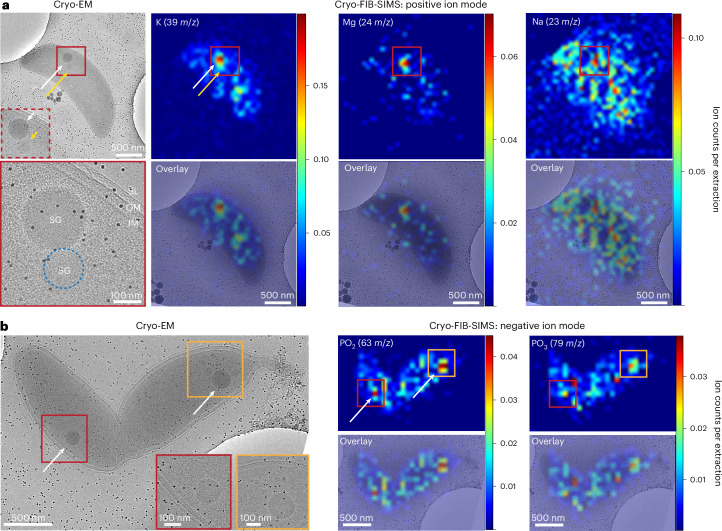
Subcellular resolution correlative cryo-EM-FIB-SIMS imaging of bacteria. **a**, Positive ion mode correlative analysis of a *C.* *crescentus* cell showing the spatial cryo-EM images at low (top) and high (bottom) magnification as well as cryo-FIB-SIMS images of three prominent *m/z* peaks (K, Mg and Na) and the respective overlays of spatial and chemical data. Ultrastructural cellular features such as the inner membrane (IM), outer membrane (OM), S-layer (SL) and storage granules (SGs) are labeled in the high-magnification cryo-EM image. For better visualization of the SGs, which are labeled by a white and yellow arrow, respectively, a zoom-in of the low-magnification image is shown in the inset (red dashed box) and the outline of the SG is enhanced in the high-magnification image (blue dashed line). The black dots are 10 nm protein-A-gold fiducials added to this otherwise untagged specimen for alignment of tilt series (cryo-ET data). **b**, Negative ion mode correlative analysis of a *C.* *crescentus* cell demonstrates the presence of phosphates within the SGs of the cell.

**Extended Data Fig. 6:**
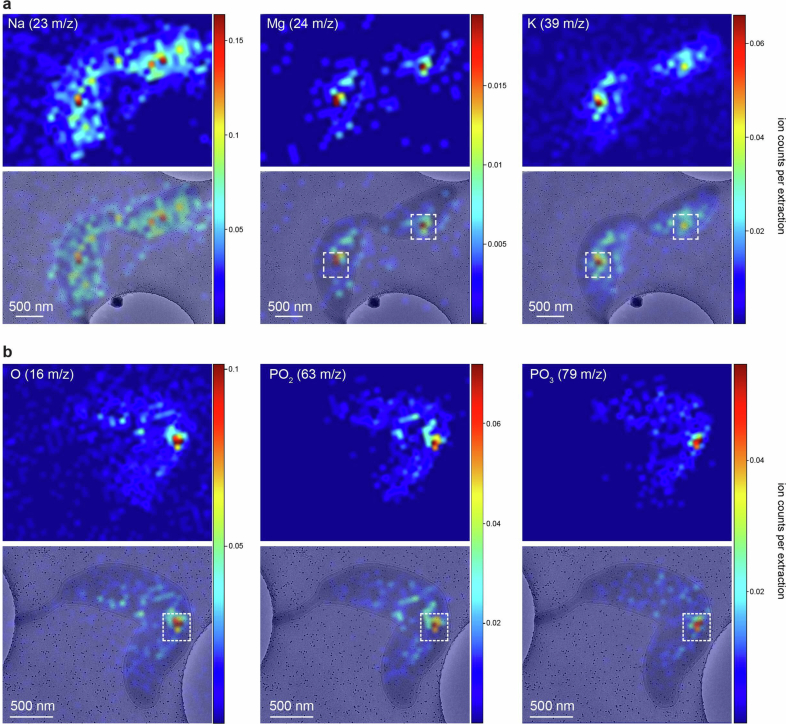
Additional examples demonstrating the cryo-EM-FIB-SIMS workflow on unlabeled *C. crescentus* cells. **a** Positive ion mode correlative analysis of a *C. crescentus* cell showing cryo-FIB-SIMS images of three prominent *m/z* peaks (Na, Mg, and K) and the respective overlays with the cryo-EM images. The dashed white boxes indicate locations of storage granules. **b** Negative ion mode correlative analysis of a *C. crescentus* cell showing cryo-FIB-SIMS images of three prominent *m/z* peaks (O, PO_2_, and PO_3_) and the respective overlays with the cryo-EM images. The dashed white boxes indicate locations of storage granules.

To understand the subcellular distribution of elements and small molecules within bacterial cells, we imaged unlabeled *C.* *crescentus* cells (Fig. 2 and Extended Data Figs. 1d and 6) by cryo-EM-FIB-SIMS. The cryo-EM images of the cells clearly show characteristic ultrastructural features, including the inner membrane, outer membrane, S-layer, as well as various intracellular features such as storage granules (Fig. 2). Overlaying the cryo-EM data with FIB-SIMS data corresponding to different *m/z* peaks and hence to different elemental and small molecular ions shows differential ionic distributions within the cell. Some elements, such as sodium (Na; 23 *m/z*), are spread across the cytosol, whereas others, for example magnesium (Mg, 24 *m/z*), are compactly localized within the storage granules. Potassium (K, 39 *m/z*), on the other hand, shows both a strong presence in the cytosol as well as in a clear, prominent peak within the storage granules (Fig. 2a). This is in line with previous studies on storage granules in different bacteria, which suggest that potassium, magnesium and calcium could act as counterions to polyphosphate chains within storage granules^23–25^. To supplement these observations about the spatial distribution of various positively charged ions, we subsequently imaged *C.* *crescentus* cells in negative ion mode. This confirmed that the storage granules are additionally rich in phosphates (PO_2_ (63 *m/z*), PO_3_ (79 *m/z*); Fig. 2b), as reported previously^26–29^.

### Cryo-EM-FIB-SIMS is compatible with cryo-LM as well as FIB-milled lamellae

**Fig. 3:**
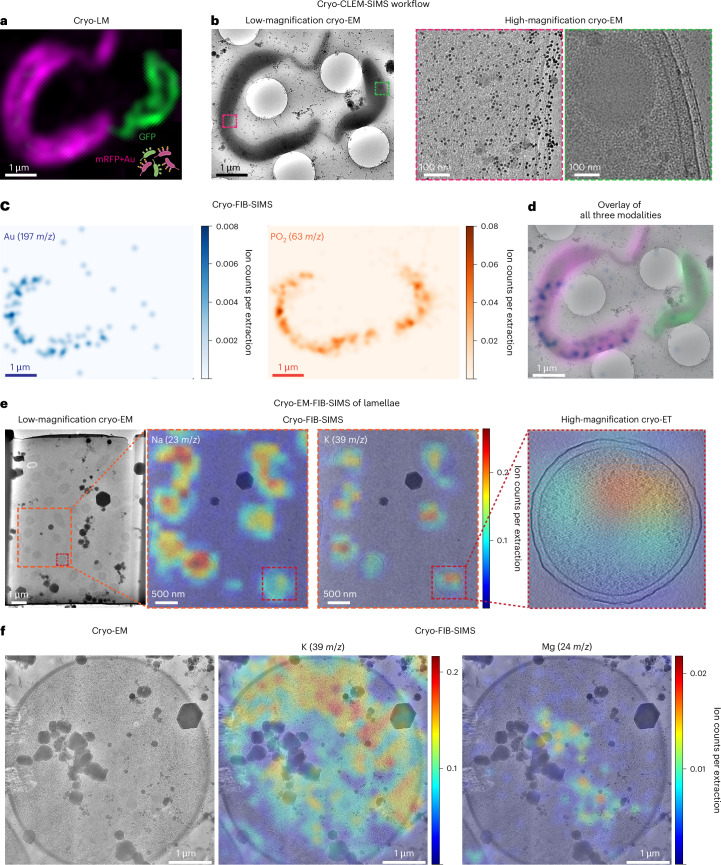
Compatibility of cryo-EM-FIB-SIMS with light microscopy workflows and thick specimen imaging. **a**–**d**, Proof-of-principle cryo-CLEM-FIB-SIMS workflow integrating cryo-LM and cryo-EM-FIB-SIMS imaging. *C.* *crescentus* cells with two types of S-layer protein labeling were prepared, resulting in a mixed population of cells with GFP labeling (green) and mRFP plus gold labeling (magenta + Au) (**a**). Imaging the same cells in cryo-EM demonstrates that the magenta-fluorescent cells feature gold nanoparticle labeling (black dots), whereas the green-fluorescent cells do not, as demonstrated both in the low-magnification image as well as in the high-magnification images of the regions marked by dashed magenta and green boxes (**b**). Cryo-FIB-SIMS only detects the magenta+Au cells in the *m/z* channel corresponding to elemental gold (left, blue) (**c**), while both cells are detected in the PO_2_ channel (right, orange), which can be further visualized by the overlay of all three imaging modalities (cryo-LM image, low-magnification cryo-EM image, cryo-FIB-SIMS image of the Au distribution) (**d**). **e**, Cryo-EM-FIB-SIMS workflow applied to cryo-FIB-milled lamellae containing *P.* *aeruginosa* bacteria and further correlated with cryo-ET of FIB-milled cells. The high-magnification cryo-ET image is a slice from a tomogram. **f**, Cryo-EM-FIB-SIMS workflow applied to cryo-FIB-milled lamellae of yeast cells shows differential signals of Mg and K between cellular compartments. Panel **a** created in BioRender; Wang, Z. https://biorender.com/z54uzfz (2026).

**Extended Data Fig. 7:**
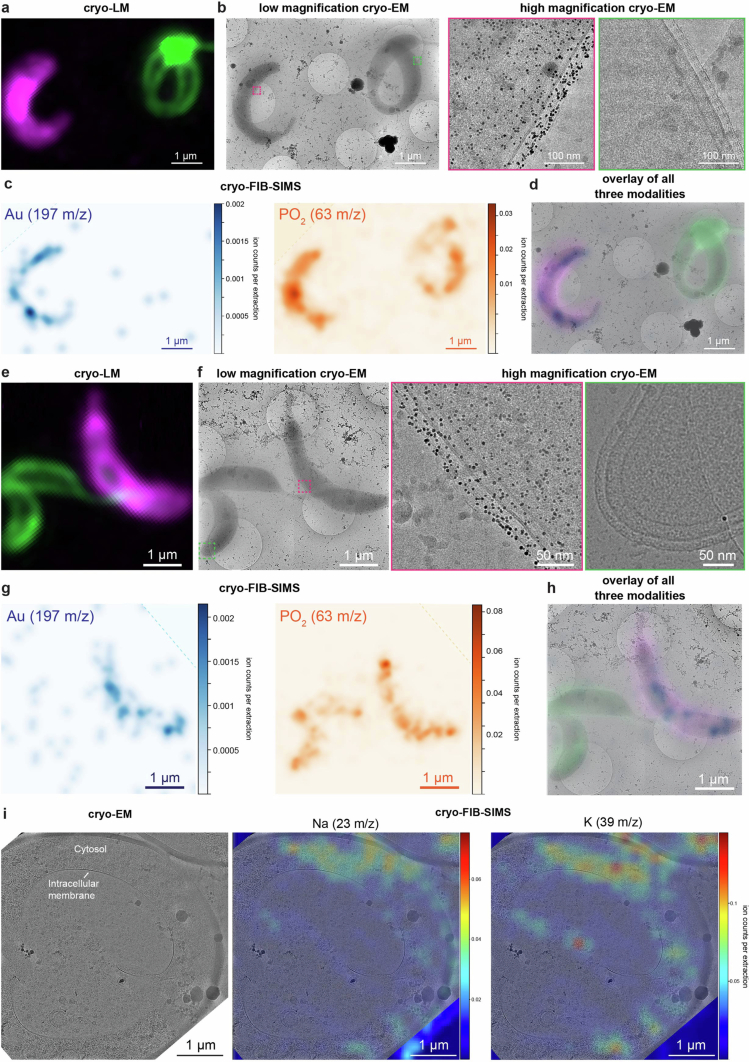
Additional examples demonstrating the cryo-CLEM-FIB-SIMS workflow as well as the applicability to eukaryotic samples. **a**, **e** Cryo-LM images showing the dual fluorescent labeling of *C. crescentus* cells. **b**, **f** Imaging the same cells in cryo-EM demonstrates that the magenta-fluorescent cells feature gold nanoparticle labeling (black dots), whereas the green-fluorescent cells do not. **c**, **g** Cryo-FIB-SIMS only detects the magenta+Au cells in the *m/z* channel corresponding to elemental gold (left, blue), while both cells are detected in the PO_2_ channel (right, orange), which can be further visualized by the overlay of all three imaging modalities (**d**, **h**). In **c** and **g**, there are missing corners of the images due to different sample rotations in the different imaging modalities, which were padded above the dashed lines to match the layout of the cryo-EM and cryo-LM images. **i** Cryo-EM-FIB-SIMS workflow applied to cryo-FIB-milled lamellae of yeast cells shows the generalizability of the workflow to eukaryotic samples.

While cryo-EM-FIB-SIMS supports spatiochemical imaging of subcellular features in bacteria, the field of view of a single measurement is small compared to light microscopy. Integrating cryo-EM-FIB-SIMS into previously established correlative light and electron microscopy (CLEM) workflows^30,31^ would thus allow large field of view imaging for target identification as well as dual labeling strategies featuring both chemical and fluorescent tags. To this end, we extended the experimental system of gold nanoparticle-tagged bacterial cells (Fig. 1b) to include differential fluorescent labeling of the S-layer protein. Using a SpyCatcher-SpyTag system for obtaining fluorescently labeled S-layers^20,32^, we labeled the S-layers either with superfolder green fluorescent protein (GFP) or monomeric red fluorescent protein (mRFP). In addition, the mRFP-labeled cells were labeled with gold nanoparticles, whereas the GFP-labeled cells were left without gold labeling (Fig. 3a). These differentially labeled cells were mixed before vitrification, resulting in a mixed population of cells that are distinguishable in all three techniques: magenta versus green fluorescence in cryo-LM (Fig. 3a), nanoparticle visibility on the cell surface in cryo-EM (Fig. 3b), and detection of gold (Au, 197 *m/z*) signal in cryo-FIB-SIMS (Fig. 3c). Imaging the same cells using all three imaging modalities demonstrates the compatibility of the full cryo-CLEM-FIB-SIMS workflow: cells that were mRFP-labeled (magenta) in light microscopy showed extensive gold labeling in both cryo-EM and FIB-SIMS imaging, whereas the green-fluorescent cells did not exhibit gold labeling as confirmed by both cryo-EM and FIB-SIMS (Fig. 3a–d and Extended Data Fig. 7a–h).

Many questions of biological interest concern eukaryotic cells, tissues or other multicellular samples, which typically require thinning before cryo-EM imaging^33^. Currently, the state-of-the-art approach for imaging these types of specimens with cryo-EM requires cryo-FIB-milling^34,35^, which produces lamellae from a thick specimen via the ablation of material above and below the target of interest. The resulting thin (100–250 nm) lamellae are suitable for imaging by cryo-EM, and specifically by electron cryotomography (cryo-ET)^1,36^, which allows 3D imaging of biological specimens. Given the importance of lamellae for cryo-ET imaging, we sought to apply cryo-FIB-SIMS imaging to lamellae. Using lamellae that were back-coated to improve stability, we imaged a specimen of concentrated *Pseudomonas* *aeruginosa* bacteria, showing clearly recognizable individual cells in the cryo-FIB-SIMS images (Fig. 3e). These images could be correlated with cryo-EM overview images as well as with high-resolution tomographic slices (Fig. 3e), demonstrating that the cryo-FIB-SIMS workflow is compatible with cryo-ET of lamellae. Next, lamellae of eukaryotic cells, namely plunge-frozen *Saccharomyces* *cerevisiae* yeast cells, were subjected to the cryo-EM-FIB-SIMS workflow (Fig. 3f and Extended Data Fig. 7i), demonstrating differential distributions of ions in different compartments of the cell. Ionic species such as K and Na showed strong localization in the cytosol across all imaged cells, whereas Mg appeared mainly within intracellular compartments. While an in-depth interpretation of these observations would require targeted experiments, these results demonstrate the generalizability of the workflow to eukaryotic cells.

### Tracking the accumulation of pollutants in environmental bacteria using cryo-EM-FIB-SIMS

Following the proof-of-principle experiments detailed above, we applied the cryo-EM-FIB-SIMS workflow to study pollutant accumulation in environmental bacteria. We studied the uptake of BPAF, a fluorinated environmental contaminant originating from plastics production, which causes endocrinal disruption in humans^37^. Gaining an understanding of the effect of BPAF exposure on organisms in the environment is an important first step toward future explorations of the impact of BPAF and related compounds on the human body as well as wider ecosystems, from both the host and host-associated microbiome perspective. Given their fluorinated nature, BPAF molecules can be tracked in our cryo-FIB-SIMS setup due to a strong fluorine signal in negative ion mode (F; 19 *m/z*), which can be used to trace the presence of fluorinated chemicals^18^.

**Fig. 4:**
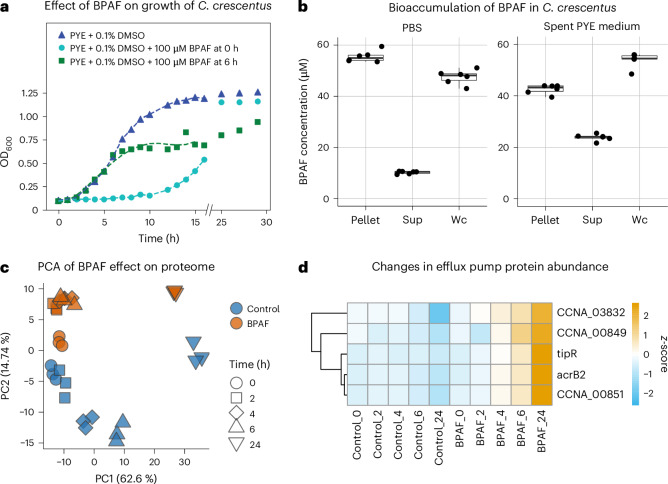
Effect of BPAF on *C.* *crescentus* cells. **a**, Growth curves of *C.* *crescentus* with (cyan and green) and without (blue) addition of BPAF. BPAF was added either at low OD_600_ at time point 0 h (cyan) or upon reaching exponential growth phase at time point 6 h (green). To ensure that the effect is due to the addition of BPAF alone, the control culture was supplemented with 0.1% dimethylsulfoxide (DMSO). PYE, peptone yeast extract. **b**, Bioaccumulation analysis performed both in PBS and spent PYE comparing BPAF concentrations between supernatant (median (PBS) 10.41 µM, median (spent PYE) 24.09 µM) and pellet (median (PBS) 54.94 µM, median (spent PYE) 43.22 µM) after exposure to 50 µM BPAF. ‘Wc’ denotes the whole culture (bacteria plus supernatant, PYE or PBS with final concentrations of 50 µM BPAF) (median (PBS) 48.12 µM, median (spent PYE) 54.49 µM), sup denotes the supernatant (PYE or PBS with 50 µM BPAF after the removal of the bacteria by spinning), and pellet denotes the cell pellet from the wc (exposed to 50 µM BPAF), reconstituted in spent medium or PBS without BPAF. For both experiments shown in this panel, sample size was *n* = 6, with each *n* corresponding to a biological replicate. Full box plot parameters are listed in the caption of Extended Data Fig. 8a. **c**, Principal-component analysis (PCA) of the effect of BPAF treatment on the proteome of *C.* *crescentus* for different exposure times. **d**, Plot of efflux pump-associated proteins with a substantial change in abundance in BPAF-exposed versus unexposed cells at incubation times 15 min (labeled 0), 2 h, 4 h, 6 h and 24 h.

**Extended Data Fig. 8:**
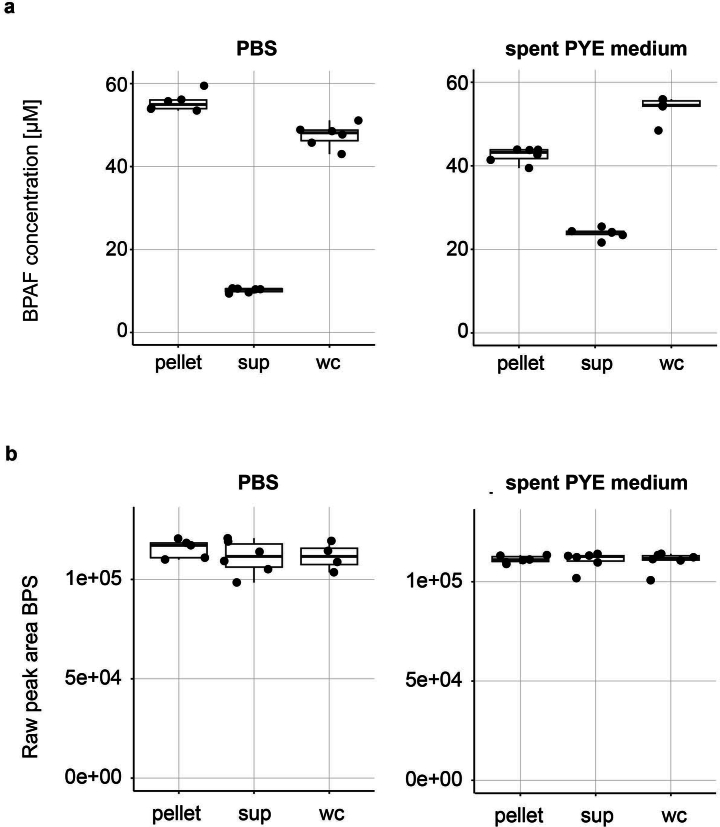
Bioaccumulation of BPAF in *C. crescentus*. Panel **a** is repeated here from Fig. 4b to facilitate comparison with the internal standard shown in panel **b**. **a** Bioaccumulation analysis performed both in PBS and spent PYE (Peptone Yeast Extract) medium shows clear differences in BPAF concentration between supernatant (median (PBS) 10.41 µM, median (spent PYE) 24.09 µM, Q1 (PBS) 9.85 µM, Q1 (spent PYE) 23.57 µM, Q3 (PBS) 10.52 µM, Q3 (spent PYE) 24.30 µM, minimum/lower whisker (PBS) 9.38 µM, minimum/lower whisker (spent PYE) 22.48 µM, maximum/upper whisker (PBS) 10.64 µM, maximum/upper whisker (spent PYE) 25.46 µM) and pellet (median (PBS) 54.94 µM, median (spent PYE) 43.22 µM, Q1 (PBS) 53.97 µM, Q1 (spent PYE) 41.47 µM, Q3 (PBS) 56.04 µM, Q3 (spent PYE) 43.84 µM, minimum/lower whisker (PBS) 53.49 µM, minimum/lower whisker (spent PYE) 39.48 µM, maximum/upper whisker (PBS) 50.46 µM, maximum/upper whisker (spent PYE) 43.89 µM) after exposure to 50 µM BPAF, indicating BPAF accumulation within the cells. wc denotes the whole culture (bacteria plus supernatant, PYE or PBS with final concentrations of 50 µM BPAF) (median (PBS) 48.12 µM, median (spent PYE) 54.49 µM, Q1 (PBS) 46.24 µM, Q1 (spent PYE) 54.26 µM, Q3 (PBS) 48.77 µM, Q3 (spent PYE) 55.54 µM, minimum/lower whisker (PBS) 43.01 µM, minimum/lower whisker (spent PYE) 52.35 µM, maximum/upper whisker (PBS) 51.09 µM, maximum/upper whisker (spent PYE) 55.94 µM), sup denotes the supernatant (PYE or PBS with 50 µM BPAF after the removal of the bacteria by spinning), and pellet denotes the cell pellet from the wc (exposed to 50 µM BPAF), reconstituted in spent medium or PBS without BPAF. The higher efficiency of bioaccumulation in PBS compared to the spent PYE medium is expected given the lack of nutrients in PBS. **b** The internal standard BPS (bisphenol S) was added after incubation as part of the extraction buffer and shows low variation in sample injection (supernatant: median (PBS) 1.12•10^5^, median (spent PYE) 1.13•10^5^, Q1 (PBS) 1.06•10^5^, Q1 (spent PYE) 1.04•10^5^, Q3 (PBS) 1.17•10^5^, Q3 (spent PYE) 1.31•10^5^, minimum/lower whisker (PBS) 9.85•10^4^, minimum/lower whisker (spent PYE) 1.06•10^5^, maximum/upper whisker (PBS) 1.21•10^5^, maximum/upper whisker (spent PYE) 1.14•10^5^; pellet: median (PBS) 1.18•10^5^, median (spent PYE) 1.11•10^5^, Q1 (PBS) 1.13•10^5^, Q1 (spent PYE) 1.10•10^5^, Q3 (PBS) 1.20•10^5^, Q3 (spent PYE) 1.13•10^5^, minimum/lower whisker (PBS) 1.10•10^5^, minimum/lower whisker (spent PYE) 1.09•10^5^, maximum/upper whisker (PBS) 1.30•10^5^, maximum/upper whisker (spent PYE) 1.13•10^5^; wc: median (PBS) 1.17•10^5^, median (spent PYE) 1.12•10^5^, Q1 (PBS) 1.10•10^5^, Q1 (spent PYE) 1.11•10^5^, Q3 (PBS) 1.27•10^5^, Q3 (spent PYE) 1.13•10^5^, minimum/lower whisker (PBS) 1.04•10^5^, minimum/lower whisker (spent PYE) 1.01•10^5^, maximum/upper whisker (PBS) 1.35•10^5^, maximum/upper whisker (spent PYE) 1.14•10^5^), demonstrating that the pattern observed in a is a biological and not a technical effect. For all experiments shown in this figure, sample size was n = 6, with each n corresponding to a biological replicate.

Exposure of the environmental bacterium *C.* *crescentus* to BPAF resulted in a dramatic retardation in growth for several hours (Fig. 4a), both when added at low optical density (OD)_600_ (0 h) and in exponential phase (6 h). Bioaccumulation analysis showed a substantial accumulation of BPAF within the bacterial cells, as demonstrated by the differences in BPAF concentration between supernatant and pellet (Fig. 4b and Extended Data Fig. 8), both in phosphate-buffered saline (PBS) and in the spent medium. The higher efficiency of bioaccumulation in PBS compared to the spent medium is expected, given the lack of nutrients in PBS. Comparison to the internal standard, bisphenol S (Extended Data Fig. 8) demonstrates that the observed pattern is a biological and not a technical effect. Proteomics characterization of cells exposed to BPAF in stationary phase for durations between 15 min and 24 h showed clear overall differences in protein abundances relative to unexposed cells at all time points (Fig. 4c). BPAF-exposed cells showed retarded proteome changes along the principal component associated with exposure time (Fig. 4c), especially at 4 and 6 h. An orthogonal difference in protein expression was observed between BPAF-exposed and unexposed cells (Fig. 4c). Specifically, we observed upregulation of several drug efflux pumps, including acrB2 and their regulatory proteins, such as TipR (Fig. 4d), indicating that the bacteria were responding to the presence of BPAF in the cytosol by attempting to pump the pollutant out; however, despite the upregulation of drug efflux machinery, bioaccumulation analysis of bacteria exposed to BPAF detected large amounts of BPAF within the bacteria (Fig. 4b).

**Fig. 5:**
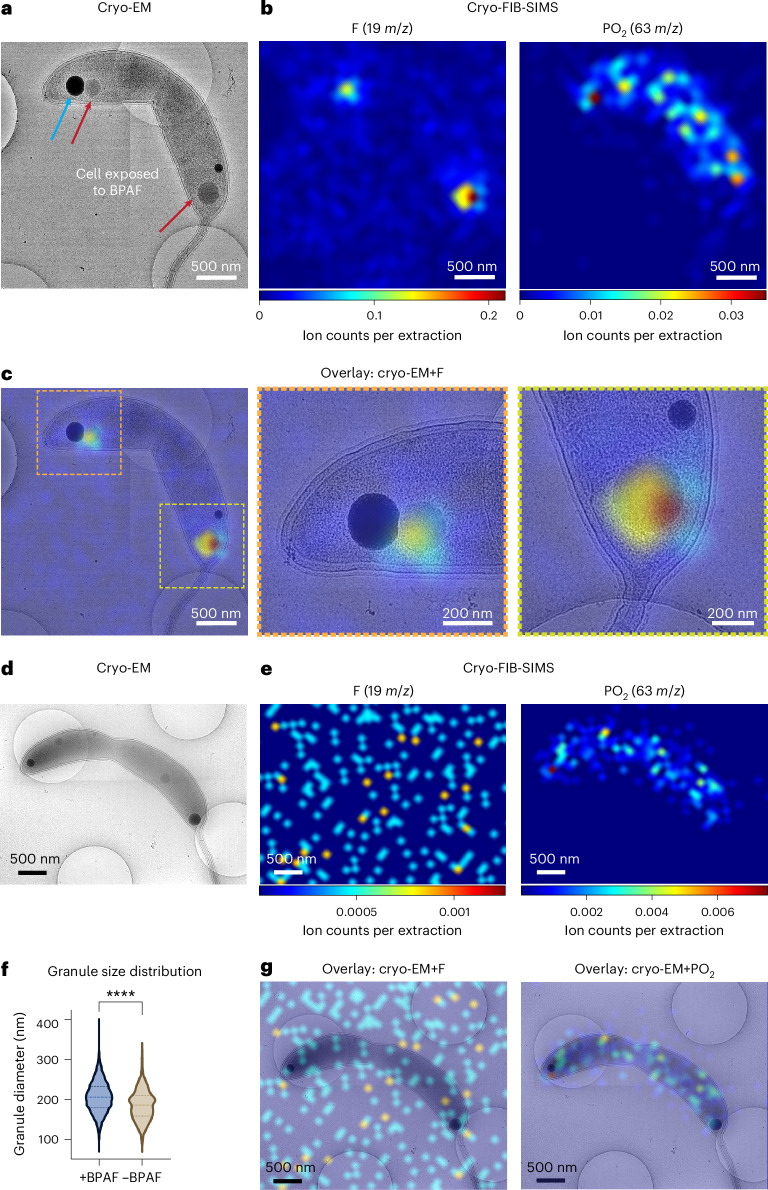
Intracellular localization of BPAF in *C.* *crescentus* cells. **a**, Cryo-EM image of a *C.* *crescentus* cell after 4 h exposure to 100 μM BPAF. Two different types of aggregates are observed within the cell: high-contrast (black) aggregates (blue arrow) and medium-contrast spherical aggregates reminiscent of storage granules (red arrows, compare with Fig. 2 and Extended Data Figs. 1 and 6). **b**, Cryo-FIB-SIMS images of the same cell as in **a** for the *m/z* peaks 19, corresponding to fluorine (left), and 63, corresponding to PO_2_ (right). **c**, Overlay of the fluorine signal and the cryo-EM image at medium magnification (left) and high magnification (right). **d**, Cryo-EM image of a *C. crescentus* cell from the same culture, grown for 4 h in PYE with 0.1% DMSO without BPAF (unexposed control sample). **e**, Corresponding cryo-FIB-SIMS signals of fluorine (F, 19 *m/z*, left) and PO_2_ (63 *m/z*, right). **f**, Comparison of the storage granule diameter in cells exposed to BPAF (left, blue) and unexposed control cells (right, beige) shows a statistically significant (***P < 0.0001, unpaired *t-*test, two-tailed) increase in granule size. **g**, Overlay of cryo-EM image and the cryo-FIB-SIMS images of fluorine (left) and PO_2_ (right).

**Extended Data Fig. 9:**
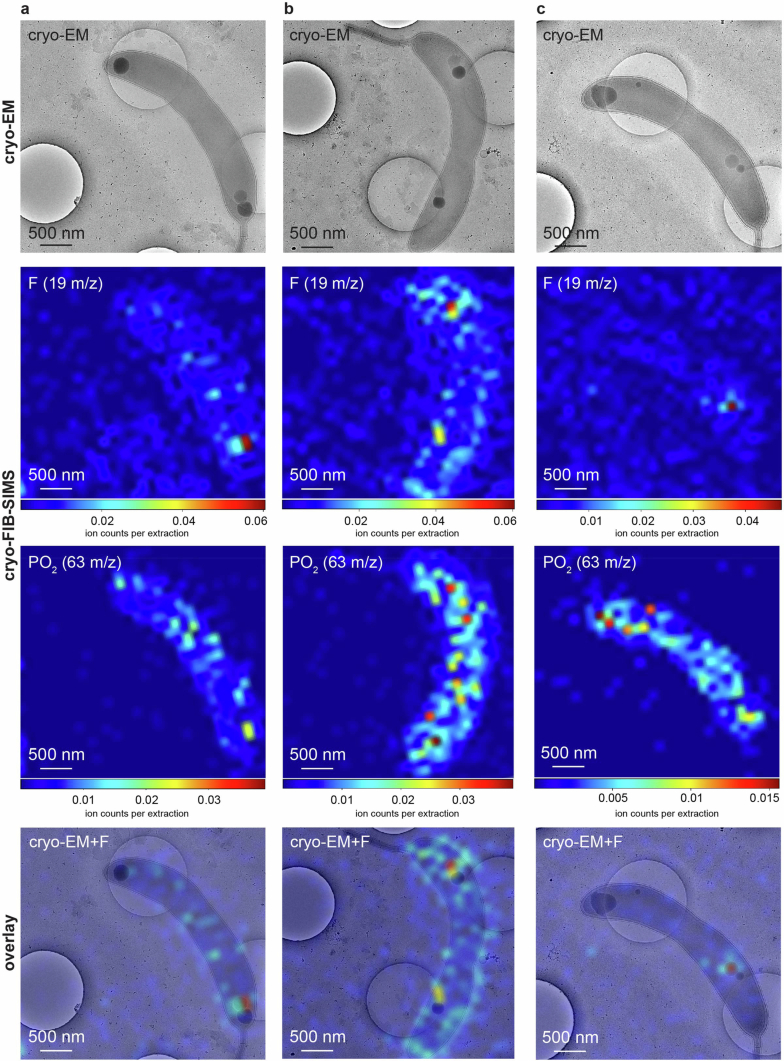
Additional examples demonstrating the uptake of BPAF by *C. crescentus*. **a**–**c** Three different *C. crescentus* cells are shown with BPAF-associated fluorine signal that is co-localized with storage granules after BPAF exposure. Top to bottom: cryo-EM images, cryo-FIB-SIMS images for F and PO_2_, overlay of the fluorine distributions with the cryo-EM images.

While bulk-level bioaccumulation analysis demonstrated uptake of BPAF by *C.* *crescentus* cells, the resulting intracellular ramifications remained unclear. To address this, we employed our cryo-EM-FIB-SIMS workflow to trace the BPAF-associated fluorine signal in the specimen and correlate it to spatial features observed in cryo-EM. Cryo-EM images of bacteria exposed to 100 µM BPAF for 4 h showed large cytosolic granular aggregates of different intensities (Fig. 5a and Extended Data Fig. 9). While the spherical, medium-contrast aggregates (Fig. 5a) are likely storage granules frequently observed in *C.* *crescentus* (Fig. 2, Extended Data Figs. 1d and 6 and Supplementary Video 2)^26–29^, the high-contrast, often irregularly shaped features (Fig. 5a, Extended Data Fig. 9 and Supplementary Video 2) are not commonly observed in *C.* *crescentus*. The cryo-FIB-SIMS imaging carried out on the same cells (Fig. 5a–c and Extended Data Fig. 9) showed a highly localized fluorine signal (Fig. 5b,c) that was not observed in the unexposed control cells (Fig. 5d,e,g), in which the fluorine signal is several orders of magnitude lower than the fluorine signal observed in BPAF-exposed cells and does not show any specific localization in the cell. This indicates that the localized fluorine signal in the treated cells is due to the presence of BPAF molecules. In contrast to the fluorine signal, other cell-associated signals, such as PO_2_, were distributed over the entire cell (Fig. 5e,g). The overlay of the cryo-EM and the fluorine cryo-FIB-SIMS images (Fig. 5c) demonstrated that the BPAF accumulation can be traced to a specific type of aggregate, namely to the storage granules, which colocalize with the fluorine signal. In contrast, the cytosol as well as the other types of aggregates do not exhibit localized fluorine signal (*n* = 162 cells, from two biological replicates). The lack of specific SIMS signal from the high-contrast granules suggests that their formation might be part of a generalized stress response from the cell.

**Extended Data Fig. 10:**
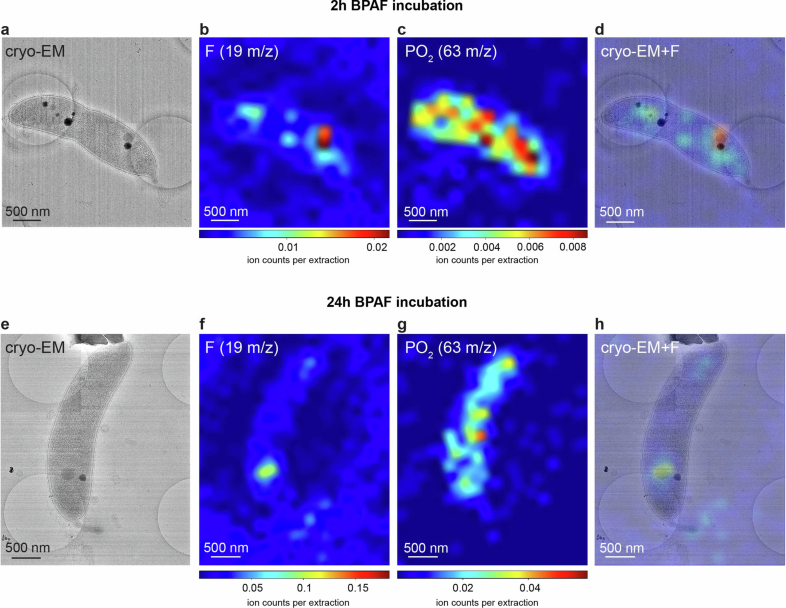
BPAF exposure at different time points. **a-d** *C. crescentus* after 2 hours of exposure to 100 μM BPAF: cryo-EM image (**a**), cryo-FIB-SIMS images for fluorine (**b**) and PO_2_ (**c**), and overlay of the fluorine distribution with the cryo-EM image (**d**) show that already after 2 hours, there is uptake and localization of BPAF in specific intracellular locations. **e**-**h** *C. crescentus* after 24 hours of exposure to 100 μM BPAF: cryo-EM image (**e**), cryo-FIB-SIMS images for F (**f**) and PO_2_ (**g**), and overlay of the fluorine distribution with the cryo-EM image (**h**) show that BPAF remains within the cells with signal localized to the storage granules in the cytosol.

While both the BPAF-exposed and the unexposed cells (Fig. 5f) show a large range of storage granule sizes, the mean diameter of the granules in the exposed cells shows a statistically significant (*P* < 0.0001) increase (206.9 ± 38.5 nm, *n* = 1,464) compared to the unexposed control cells (186.4 ± 37.9 nm, *n* = 252). These results strongly suggest that BPAF is sequestered in the granules after uptake by the cells, thus removing BPAF from the cytosol. In conjunction with the upregulation of drug efflux machinery, the sequestration of BPAF in the granules suggests that BPAF cannot be efficiently removed from the cell. This is in line with similar observations in *Escherichia* *coli* ΔtolC mutants described previously^18^ for other fluorinated compounds, indicating that sequestration inside aggregates could be an alternative way of lowering pollutant concentration in the cytosol. Imaging BPAF-exposed cells at both shorter (2 h) and longer exposure times (24 h) showed strongly localized intracellular aggregation of BPAF at both time points (Extended Data Fig. 10), implying that the pollutant is taken up quickly and retained within the cells for a long period of time. Cryo-EM-FIB-SIMS imaging thus allowed us to pinpoint the precise localization of BPAF accumulation within *C.* *crescentus* cells, yielding insights into how these cells accumulate toxic environmental pollutants at the subcellular level.

## Discussion

We report a cryo-EM-FIB-SIMS workflow (Figs. 1 and 2) that can be applied in a modular way to a variety of samples, including untagged bacteria (Fig. 2), fluorescently tagged bacteria (Fig. 3a–d), and eukaryotic specimens (Fig. 3f). The versatility of the workflow, achieved by the combination of various imaging modalities, opens up a wide range of future applications, such as the subcellular (sub-organellar in eukaryotes) localization of molecules of interest or the targeted exploration of local morphology and chemical composition of subcellular features in both prokaryotic and eukaryotic cells. Using the full cryo-CLEM-FIB-SIMS workflow will be especially beneficial when studying thicker and more complex samples, such as eukaryotic cells or multicellular tissue-like specimens, as fluorescence can be used to guide lamellae milling as well as site-specific cryo-EM and cryo-FIB-SIMS imaging.

Labeling strategies using both mass spectrometry-accessible labels such as metals (Fig. 1b) or isotopes (Extended Data Fig. 5c), as well as orthogonal fluorescent labeling, could be used for targeting and tracking larger molecules. While cryo-FIB-SIMS imaging itself can provide depth information, volumetric cryo-EM-FIB-SIMS imaging could be further developed by exploiting the compatibility of the workflow with cryo-ET as well as 3D cryo-FIB-SEM (serial block face volumetric imaging). In this report, we used our cryo-EM-FIB-SIMS to study the outstanding question of the uptake of fluorinated contaminants by environmental bacteria, demonstrating the power of correlative spatiochemical imaging for uncovering mechanistic biology.

These results anchor cryo-EM-FIB-SIMS within the spectrum of spatiochemical imaging techniques for biological specimens, which ranges from other SIMS-based methods to spectroscopic techniques such as electron energy loss spectroscopy and energy dispersive X-ray spectroscopy^15,16,38–43^. While these methods all generate spatially resolved information about chemical composition and molecular localization, they differ vastly in compositional sensitivity and spatial resolution. As SIMS directly detects the secondary ions produced during the interaction between primary ion beam and sample, not only elemental, but also molecular and isotopic secondary ions can be detected. The detectable mass range depends on the primary ion beam in the first instance, and on the mass resolution and the number of simultaneously detectable ions, which are characteristics of the mass spectrometer, in the second instance. Electron energy loss spectroscopy, energy dispersive X-ray spectroscopy and scanning transmission electron microscopy detect scattered electrons or photons, hence only data regarding elemental composition, but not about molecules or isotopes, is acquired, making cryo-FIB-SIMS complementary to those methods that offer higher spatial resolution up to the sub-molecular range.

The attainable spatial resolution in SIMS depends on the choice of primary ion beam and the ionization characteristics (including sputter yield) of the secondary ions being considered. Using monoatomic primary ion beams such as Ga permits high spatial resolution SIMS imaging, allowing the resolution of features up to approximately 50 nm (refs. ^6,44^). By combining SIMS imaging with low-dose, high-resolution spatial imaging techniques such as cryo-EM and cryo-SEM^16,45^ in sequential imaging workflows, the chemical and spatial data can be correlated to enable the nanometer scale localization of elements and molecules within the cellular environment. While cryo-SEM imaging offers compatibility with volumetric imaging on thicker samples, workflows employing cryo-EM or cryo-ET, as presented here, allow correlative imaging at unprecedented spatial resolution at which individual molecules can be resolved. Larger molecules can be tracked in cryo-FIB-SIMS imaging via their elemental (for example, metals and halogens) or small molecular degradation fragments (Supplementary Table 1) in untagged or labeled specimens^12,21,22,46^; however, the low ionization yield as well as the large interaction depth of the Ga beam with the sample^47^ results in a very low probability of extracting peptides, lipids or oligonucleotides. To extend the range of detectable molecules, larger ion beams^9,11,12^ such as gas cluster ion beams^4^ could be utilized, which would have the added benefit of very precise depth analysis, albeit at the expense of lateral spatial resolution. Further development of the method to encompass correlative cryo-EM and SIMS imaging with a range of different primary ion beams could, in the future, provide comprehensive data on ions of a larger mass range, including peptides, lipids and drug molecules, while retaining the high spatial resolution characterizing the technique described here. We suggest that combinations of techniques with high spatial and chemical resolution, including cryo-EM-FIB-SIMS and other recently reported methods^15,45,48–51^ will be important to obtain a comprehensive molecular understanding of complex biological specimens.

## Methods

### Sample preparation

#### Preparation of CsI and H_2_O samples for cryo-FIB-SIMS

Vitrified samples of CsI and H_2_O were prepared by applying 2.5 µl of saturated CsI solution (in water) and deionized water, respectively, to cryo-EM grids (Quantifoil Cu/Rh R3.5/1), followed by blotting from both sides in a Vitrobot Mark IV (Thermo Fisher) and plunge-freezing in liquid ethane which was maintained below −170 °C. The chamber was kept at 100% relative humidity at a temperature of 10 °C, with blotting conditions comprising a blot time of 5 s, blot force of −10 and wait time of 2 s.

#### Unlabeled *C.crescentus* for cryo-EM-FIB-SIMS analysis

*C.* *crescentus* cells (strain CB15N) were inoculated from a frozen stock into 5 ml of PYE (peptone yeast extract) medium (0.2% (w/v) bactopeptone, 0.1% (w/v) yeast extract, 0.5 mM CaCl_2_, 1 mM MgSO_4_)^52^ and grown in a shaking culture at 30 °C to an OD_600_ of 1. Subsequently, cells were prepared for cryo-EM imaging^53^. In brief, 2.5 µl of the cell culture was applied to a cryo-EM grid (Quantifoil Cu/Rh R1.2/1.3), blotted from both sides in a Vitrobot Mark IV (Thermo Fisher) and plunge-frozen into liquid ethane which was maintained below −170 °C. The chamber was kept at 100% relative humidity at a temperature of 10 °C, with blotting conditions comprising a blot time of 2.5 s, blot force of −7 and wait time of 30 s.

#### Fluorescent and gold nanoparticle-labeled *C.crescentus* for cryo-CLEM-FIB-SIMS analysis

##### Purification of His-tagged SpyCatcher fusion proteins

For purification of His-tagged SpyCatcher fusion proteins^20,32^, plasmids pDEST14-SpyCatcher-superfolder GFP (sfGFP) and pBAD-SpyCatcher-mRFP1 were transformed (separately) into *E.* *coli* BL21 DE3 cells and grown in LB in the presence of 100 μg ml^−1^ ampicillin. For each construct, a 6-l culture was grown at 37 °C with shaking until an OD_600_ of 0.8 was reached. The culture expressing SpyCatcher-sfGFP was induced with 0.5 mM IPTG (isopropyl β-D-1-thiogalactopyranoside); while the culture expressing SpyCatcher-mRFP1 was induced with 0.1 % (w/v) arabinose for 16 h at 18 °C. Cells were pelleted at 4,000*g* (relative centrifugal force) for 30 min at 4 °C and resuspended in 150 ml lysis buffer (50 mM HEPES(4-(2-hydroxyethyl)-1-piperazineethanesulfonic acid)/NaOH, pH 7.5, 500 mM NaCl, 1 mM MgCl_2_, 5% glycerol, 50 µg ml^−1^ DNase I, 50 µg ml^−1^ RNaseA, 0.2 mM TCEP (tris(2-carboxyethyl)phosphine) and two Complete Protease Inhibitor tablets (Roche)). Cells were passed through a homogenizer at a pressure of 20,000 psi (pounds per square inch) for two cycles. Cell debris was pelleted at 20,000*g* for 30 min at 4 °C. The supernatant was clarified by passing through a 0.22-μm filter and loaded onto a 5-ml HisTrap column (Cytiva). Elution was performed by applying the elution buffer (50 mM HEPES/NaOH, pH 7.5, 500 mM NaCl, 5% glycerol, 0.2 mM TCEP and 500 mM imidazole) with a shallow gradient. The fractions containing the SpyCatcher conjugates were pooled and dialyzed overnight into the gel filtration buffer (50 mM HEPES/NaOH, pH 7.5, 150 mM NaCl, 5% glycerol and 0.2 mM TCEP). The protein solution was concentrated to a 5 ml volume and then loaded onto a HiLoad Superdex S200 16/600 column (Cytiva). The eluted peaks were pooled, flash-frozen in liquid nitrogen and stored at −80 °C until further use.

##### *Ccrescentus* labeling with SpyCatcher fusion proteins and NanoGold

*C.* *crescentus* cells (CB15N Δ*sapA rsaA*467:SpyTag) were grown in PYE medium at 30 °C with shaking at 180 rpm. Cells grown to exponential growth phase were diluted to an OD_600_ of 0.1 in a total culture volume of 250 μl and mRFP-SpyCatcher was added to final concentration of 8 μM. The labeling mixture was incubated at 23 °C for 90 min. After the incubation, cells were washed three times with PYE and pelleted at 6,000*g* for 3 min at 23 °C. Cells were resuspended into 105 μl PYE after the final wash, to which 105 μl of 5 nm-Ni-NTA (nickel (II) nitraloacetic acid) NanoGold (Nanoprobes) was added to achieve a final 0.25 μM concentration of 5 nm Ni-NTA-NanoGold. The final NaCl concentration was brought to a more physiologically relevant range of 120 mM. After this, the gold labeling reaction was commenced by incubation for 30 min at 23 °C. After incubation, cells were washed three times with PYE and resuspended in fresh 5 μl PYE. In parallel, cells labeled with sfGFP-SpyCatcher were prepared using the same procedure except for the omission of the gold labeling reaction. In brief, cells grown to exponential phase were diluted to an OD_600_ of 0.1 with a final volume of 250 μl and sfGFP-SpyCatcher was added to a final concentration of 8 μM and incubated for 90 min at 23 °C. Cells were washed three times, and the final resuspension was performed into 5 μl of PYE. Before plunge-freezing for cryo-EM, mRFP-SpyCatcher-Nanogold and sfGFP-SpyCatcher-labeled cells were mixed in a 2:1 ratio.

Cryo-EM grids were prepared as described previously^54^. In brief, 3.5 µl of the resuspended sample was applied to a freshly glow-discharged 135 mesh Quantifoil London Finder grid (H15) and plunge-frozen into liquid ethane maintained at −178 °C, using a Vitrobot Mark IV at 10 °C and relative humidity of 95% after a wait time of 30 s, with −2 blot force, 0.5 s drain time and 2.5 s blot time.

#### Isotope-labeled *B. thetaiotaomicron*

*B.* *thetaiotaomicron* cultures were grown at 37 °C in an anaerobic chamber (Coy Laboratory Products) under a gas mix of 2% H_2_ and 12% CO_2_ in N_2_. Frozen stocks of *B.* *thetaiotaomicron* VPI-5482 (DSM 2079) were precultured overnight in modified Gifu anaerobic medium (mGAM; HyServe). Cells were collected by centrifugation (5 min, 5,000*g*), washed in PBS, and inoculated at OD_600_ 0.05 into M9 minimal medium supplemented with 50 mg l^−1^ L-cysteine, 5 mg l^−1^ hemin, 2.5 µg l^−1^ vitamin K_1_, 2 mg l^−1^ FeSO_4_·7H_2_O and 5 µg l^−1^ vitamin B_12_. Cultures contained either 1 g l^−1^ ^13^C-labeled starch or 1 g l^−1^ glucose. After 24 h of growth, cells were collected (5 min, 5,000*g*) and washed twice in PBS before cryo-EM-FIB-SIMS analysis. Subsequently, cells were prepared for cryo-FIB-SIMS imaging. In brief, 2.5 µl of cell culture was applied to a cryo-EM grid (Quantifoil Cu/Rh R2/2), blotted from both sides in a Vitrobot Mark IV (Thermo Fisher) and plunge-frozen into liquid ethane which was maintained below −170 °C. The chamber was kept at 100% relative humidity at a temperature of 10 °C, with blotting conditions comprising a blot time of 2.5 s, blot force of −7 and wait time of 30 s.

#### High-pressure frozen *P.aeruginosa* for cryo-EM-FIB-SIMS of lamellae

*P.* *aeruginosa* (strain PAO1) cells were cultured overnight on an LB agar plate. Bacterial cells were collected by scraping off the plate and resuspended in LB medium with 5% glycerol to reach an OD_600_ of around 100. Cells were vitrified on an EM grid by high-pressure freezing using a Compact 03 HPF (M. Wohlwend) following the ‘waffle’ method protocol^55^. In brief, 2 µl of sample was applied to a Quantifoil holey carbon grid (R2/2 mesh 200), which was then sandwiched between the flat sides of two metal planchettes (3-mm diameter, 0.5-mm thickness). This assembly was inserted into a HPF holder (depth 0.95 mm) for freezing.

#### Plunge-frozen yeast for cryo-EM-FIB-SIMS of lamellae of eukaryotic cells

*Saccharomyces* *cerevisiae* (strain BCY-123) yeast cells were cultured overnight at 30 °C in yeast extract peptone medium supplemented with 2% glycerol. The culture was adjusted to OD_600_ of 0.6. Cells were vitrified on glow-discharged Quantifoil Au + SiO_2_ R1.2/1.3, 200 mesh grids after backside blotting with blot time of 4–5 s using an EM GP2 Leica plunger at 22 °C and 99% relative humidity.

#### Sample preparation for studying BPAF uptake by *C.crescentus* using cryo-EM-FIB-SIMS and proteomics

*C. crescentus* cells were inoculated from a frozen glycerol stock culture into 30 ml PYE medium and grown overnight at 30 °C. The resulting pre-culture was then used to inoculate three flasks with 400 ml PYE medium (10 ml per flask) and grown for 24 h. The culture was then centrifuged at 4,500*g* for 10 min to pellet the cells. The cells were next resuspended into the same medium and adjusted to an OD_600_ of 3.75 for cryo-EM-FIB-SIMS imaging and to an OD_600_ of 2 for proteomics studies. For cryo-EM-FIB-SIMS, the culture was split into two flasks containing 30 ml of culture each. To one of the two flasks, a stock solution of BPAF in DMSO was added resulting in a final concentration of 100 µM BPAF and 0.1% (v/v) DMSO. To the other flask, serving as a control sample, 0.1% (v/v) DMSO was added. Both flasks were incubated at 30 °C for 4 h and two independent biological replicates were analyzed. The cells were prepared for cryo-EM analysis as described in the previous section for unlabeled cells. For proteomics, the culture was split into six flasks of 30 ml culture each. To three of them, BPAF in DMSO was added to achieve a final concentration of 100 µM BPAF and 0.1% (v/v) of DMSO, and the control samples were adjusted to a final concentration of 0.1% (v/v) DMSO. Samples were collected after 15 min and after 2, 4, 6 and 24 h in a standing incubation at 30 °C. Three ml of each of the samples were centrifuged at 16,800*g* for 1 min and the pellet was directly frozen at −80 °C.

#### Bioaccumulation analysis of BPAF in *C.crescentus*

##### *C.crescentus* growth and treatment

*C.* *crescentus* cells were grown overnight in PYE medium, the culture was split, centrifuged at 4,500*g* for 10 min, and the pellets were washed once with PBS and spent PYE medium (the medium in which the cells were grown in), respectively, and subsequently resuspended at an OD_600_ of 3.75 in the respective medium (PBS or spent PYE). Then, 50 µM BPAF were added to the samples from a 50 mM stock solution in DMSO. The samples (six replicates, three DMSO controls and four incubation controls without bacteria) were incubated at 30 °C for 4 h.

##### Sample collection and extraction for BPAF bioaccumulation

Whole culture was collected and the remaining sample was centrifuged for 2.5 min at 30 °C at 16,900*g*. Supernatants were collected and the pellets were resuspended in equivalent amount of BPAF-free PBS and spent PYE medium, respectively. All samples were extracted by adding 4× the amount of ice cold (−20 °C) extraction buffer (1:1 ratio of methanol and acetonitrile with internal standards, 50 µM bisphenol S (for negative mode), spiked in) followed by an incubation on ice for 15 min. Samples were then centrifuged at 4 °C at 16,900*g* for 2.5 min and supernatants were transferred to vials for liquid chromatography–mass spectrometry (LC–MS) analysis. Samples for concentration calibration (1:2 dilution of BPAF) and bacteria-free compound controls were processed in the same way.

##### LC–MS measurements for BPAF bioaccumulation

Analysis was performed on an Agilent 1290 Infinity II LC system coupled with an Agilent 6470 triple quadrupole mass spectrometer with JetStream ESI source operated in dynamic multiple reaction monitoring mode. BPAF and bisphenol S were detected in negative ion mode. Chromatographic separation was performed using a ZORBAX RRHD Eclipse Plus column (C18, 2.1 × 100 mm, 1.8 μm; Agilent 858700-902) at 40 °C. The multisampler was maintained at a temperature of 4 °C. The injection volume was 1 μl and the flow rate was 0.4 ml min^−1^. The mobile phases consisted of A: water + 0.1% formic acid + ammonium formate 10 mM; B: acetonitrile + 0.1% formic acid + ammonium formate 10 mM. The 10-min gradient started with 35% solvent B, which was increased to 100% by 9 min and held for 1 min before returning to 35%. A pooled quality control sample, blanks and serial dilutions of BPAF were injected regularly throughout each run. Data were analyzed using MassHunter Workstation Quantitative Analysis for QQQ v.10.1. Concentrations were obtained via external calibration using standard curves.

### Imaging methods

#### Cryo-FIB-SIMS

Cryo-FIB-SIMS measurements were carried out on a gallium (Ga) FIB-SEM (Zeiss Crossbeam 550) equipped with a ToF mass spectrometer (TOFWERK fibTOF). During cryo-FIB-SIMS imaging, the region of interest was scanned by the gallium FIB, whose interaction with the sample results in the ablation of material at each pixel. The resulting secondary ions are extracted into and analyzed by the ToF mass spectrometer at each pixel. As a full mass spectrum is produced at each pixel, the chemical composition of the sample can be visualized by plotting the pixel-by-pixel intensity for each detected mass-to-charge ratio. The range of detectable mass-to-charge ratios depends on the dwell time of the FIB at each pixel. For the standard settings used here (FIB dwell time per pixel 12.8 μs), this corresponds to a *m/z* range from 1 to 323. The image intensity (color scale) maps the ion counts per extraction (one extraction corresponding to one pixel in FIB scanning; Extended Data Fig. 1). Simultaneously, secondary electrons resulting from the interaction of the FIB and the specimen can be detected, yielding spatial images of the sample (FIB images). Depending on the polarity of the extraction voltage, either positively or negatively charged secondary ions can be extracted and detected.

The imaging geometry is of the cryo-FIB-SIMS instrument is depicted in Extended Data Fig. 2. The FIB column and the ToF mass spectrometer have nearly the same angular orientation with respect to the sample. While the cryo-stage (Quorum) can be rotated by 360° and allows tilting as well as *xyz* motion, thus enabling imaging in a variety of geometries, the best ion extraction can be achieved if the sample plane is perpendicular or near-perpendicular to the ToF axis. During FIB-SIMS imaging, the ToF extraction optics are inserted close to the sample (Extended Data Fig. 2b) to allow the application of the extraction voltage near the sample, resulting in an orthogonal deflection of the ions into the ToF analyzer. During the imaging process, the SIMS control software (TofDaq and TOFWERK) controls the FIB scan generator to align the FIB scanning with the extraction pulses.

As the imaging process removes material from the sample, repeated scanning of the area of interest yields 3D information of the sample composition as each 2D dataset can be regarded as a slice of the 3D sample volume analogous to a ‘*Z*’-stack in light microscopy, where the ‘*Z*’ axis is the ion beam direction. The resulting volume hence allows both extraction of ‘*X*–*Y*’ view images (frames) orthogonal to the ion beam direction as well ‘*X*–*Z*’ or ‘*Y*–*Z*’ slices along the direction of the ion beam for visualizing the variation of sample composition with depth (Extended Data Fig. 4 and Supplementary Video 1).

The penetration depth depends on various factors such as beam current, voltage and dwell time. If the samples are homogeneous, homogeneous ablation should be achieved; however, due to different features within the sample interacting differently with the primary ion beam as well as beam damage artifacts, inhomogeneities during ablation can occur.

For cryo-FIB-SIMS imaging, the Ga beam was operated at 30 kV and 50 pA, the scanning area was 512 × 512 pixel with MS data collected using a binning of 8 × 8 pixels, an FIB dwell time per pixel of 12.8 µs, extraction pulse with of 1,000 ns and magnification-dependent unbinned pixel sizes ranging from 10–30 nm. The depth of material removed by a single scan (corresponding to one frame) depends on beam current, pixel size and dwell time. For the beam settings reported here, a rough estimate using the sputtering rate of Ga beam milling on vitrified ice^56^ yields a depth per frame of 10–50 nm. The mass resolution of the instrument is specified as >700 by the manufacturer and was measured to be in the range of 1,000–1,500 in the experiments performed during this study.

Analysis of the cryo-FIB-SIMS data was carried out using the TofSimsExplorer v.1.15.2.0 (Tofwerk) and Python v.3.9.14.

To overlay cryo-EM and cryo-FIB-SIMS data, we scale the cryo-EM data and the FIB-SIMS data (which includes both the spatial FIB image from secondary electron detection and the SIMS data) to the same size. As spatial features are generally more clearly discernible in the secondary electron FIB image, we manually overlay the cryo-EM and the FIB images, using larger features such as the cellular outline and larger intracellular aggregates as well as the holes in the carbon support film. As the FIB and SIMS images are collected simultaneously and with the same field of view and pixel size, the subsequent overlay with the SIMS data is straightforward.

#### Sample backside coating for enhanced stability

For compatibility with cryo-EM imaging, specimens need to be prepared on electron-transparent supports such as grids with amorphous carbon films. In principle, cryo-FIB-SIMS imaging can be directly carried out on these specimen supports^18^; however, for increased stability and reduced sample movement, which increases both the overall MS imaging quality and specifically the signal intensity from holey supports (Extended Data Fig. 3), we found it beneficial to apply an organometallic coating before cryo-FIB-SIMS imaging. For this, we first sputtered the back of the sample (facing away from the ion beam during cryo-FIB-SIMS imaging) with a thin layer of platinum in the transfer load lock (Quorum) and then applied an organometallic coating (C_9_H_16_Pt) to the back of the sample using the gas injection system in the FIB-SEM chamber (Zeiss).

#### Cryo-EM data collection

The 2D cryo-EM data were collected on either a Titan Krios G1 or a Glacios microscope (Thermo Fisher) operating at an acceleration voltage of 300 kV or 200 kV, respectively, equipped with a direct electron detector (Gatan/Thermo Fisher) using EPU 2 software (Thermo Fisher). Medium-magnification (full cell) images were recorded at nominal magnifications ranging from ×6,500–11,500, corresponding to pixel sizes of 23.89 to 18.01 Å, at exposure times of 2–5 s. High-magnification images were recorded at ×42,000 magnification (pixel size of 1.856 Å). Cryo-ET data were collected on a Titan Krios G3 microscope (Thermo Fisher) operating at an acceleration voltage of 300 kV, fitted with a Quantum energy filter (slit width 20 eV) and a K3 direct electron detector (Gatan) using SerialEM 3 software. Cryo-ET tilt series were collected using a grouped dose-symmetric tilt scheme as implemented in SerialEM^57^, with a total dose of 120 electrons/Å^2^ per tilt series, nominal defocus of −8 μm, and with ±60° tilts of the specimen stage at 1° tilt increments. Tilt series images were collected using a physical pixel size of 3.42 Å.

#### Cryo-LM

Fluorescence microscopy images of the vitrified cells were acquired using a Zeiss LSM 900 confocal microscope equipped with an Airyscan 2 detector, Axiocam 306 camera, a Colibri 5 LED light source (Zeiss Microscopy) and a CMS196V3 (serial number DV5260-0009) cryo-stage (Linkam Scientific). Vitrified grids were initially inspected using a ×0.5/0.2 NA objective and high-resolution images were obtained using an LD EC Epiplan-Neofluar ×100/0.75 NA objective. Due to variable ice thickness, cells were positioned at different depths within the specimen and therefore ‘*Z*’-stacks of 2-μm thickness with 0.5-μm intervals were acquired. Airyscan processing was performed for all high-resolution images in the Zen software (Zeiss Microscopy). When necessary, image registration was applied using Linear Stack Alignment with the SIFT plugin^58^. Maximum intensity projections were performed on the *Z*-stacks using Fiji (ImageJ v.2.16.0)^59^.

#### Lamella preparation, cryo-ET data acquisition and visualization

Lamellae of *P.* *aeruginosa* cells were prepared using a Crossbeam 550 (Carl Zeiss) equipped with a Quorum cryo-stage using SmartFIB software (v.1.15) following the ‘waffle’ milling strategies reported previously^55^. The grid was first sputter-coated with platinum for 45 s (5 mA) followed by coating with organic platinum through the gas injection system for 60 s. Initial trenches of 30 μm × 40 µm on both sides of the targeted regions were milled perpendicular to the grid with 30 nA FIB current. The targeted regions were gradually thinned down to 5 µm thickness using 7 nA, 3 nA and 1.5 nA FIB currents at a milling angle of 20 °. Following the milling of a stress-release notch, the final lamellae were produced in four steps using currents of 700 pA, 300 pA, 100 pA and 50 pA. Low-magnification images (at ×2,300 (pixel size 39.23 Å) or ×8,700 (pixel size 10.26 Å) nominal magnification), as well as tomographic data were acquired in a Titan Krios G3 (Thermo Fisher Scientific) equipped with a K3 summit energy filter (Gatan) using SerialEM3 software. Tilt series were acquired at ×42,000 nominal magnification (pixel size 2.13 Å) with a dose-symmetric tilt scheme (3° increment, −54° to +54° tilt range relative to the lamella plane). Tomograms were reconstructed using the AreTomo3^60^ software and visualized using the IMOD (v.5.1) software^61^. Cryo-FIB milling of the yeast cells was performed using an Aquilos2 dual beam FIB-SEM (FIB-scanning electron microscope, Thermo Fisher). The grids were sputter-coated with platinum (0.1 mbar; 30 mA, 1 V, 30 s), then mapped using the MAPs software (v.3.27; Thermo Fisher). A coating of organic platinum was applied via the gas injection system for 20 s, followed by a final sputter-coat, using the settings described above. AutoTEM (v.2.4.3; Thermo Fisher) was set to rough mill the lamellae in three steps with currents of 0.5 nA, 0.3 nA and 0.1 nA with a milling angle of 10°. Lamellae polishing was performed in two steps at currents of 50 pA and at 30 pA to produce lamellae with a final thickness of approximately 200 nm. Cryo-EM images of the lamellae at ×8,700 nominal magnification (pixel size 10.26 Å) were acquired in a Titan Krios G3 (Thermo Fisher Scientific) equipped with a K3 summit energy filter (Gatan) using SerialEM3 software.

#### Statistics and reproducibility

The following numbers of cells were analyzed in the cryo-EM-FIB-SIMS experiments shown in this study. All sample size numbers refer to fully correlated datasets (they include both cryo-EM micrographs as well as cryo-FIB-SIMS data (and, where applicable, cryo-LM images) for each of the analyzed cells): gold nanoparticle-labeled *C.* *crescentus* (Fig. 1b) *n* = 24 on two separately prepared grids; unlabeled *C.* *crescentus* (Fig. 2): *n* = 105 on three grids from separately prepared cultures; isotope analysis (Extended Data Fig. 5c): *n* = 22 *B.* *thetaiotaomicron* cells incubated in ^13^C-labeled starch, *n* = 13 in control (glucose) from two biological replicates; cryo-CLEM-FIB-SIMS: mRFP + Au-labeled (Fig. 3): *n* = 24, GFP-labeled: *n* = 29 from two separate labeling experiments; BPAF uptake (Fig. 5): *n* = 162 BPAF-exposed, *n* = 50 unexposed imaged on a total of eight grids from three biological replicates. Storage granule size measurements were carried out using Fiji (ImageJ v.2.16.0)^59^ from cryo-EM images for *n* = 1,464 granules in BPAF-exposed cells, *n* = 252 in unexposed cells, and statistical tests (unpaired *t*-test, two-tailed) were performed using GraphPad Prism v.10.4.1. For the cryo-FIB-SIMS experiments on lamellae, 15 lamellae of concentrated *P.* *aeruginosa* and 20 lamellae of yeast cells were imaged from two independent biological replicates.

#### Proteomics

For MS analysis, total protein extracts were prepared by lysing cells in 20 mM HEPES containing 0.2% RapiGest (Waters, w/v). Nucleic acids were digested by incubating the cell lysates with 500 U of Benzonase nuclease (Sigma) for 30 min at room temperature. Cysteines were reduced by adding dithiothreitol to a final concentration of 4 mM and then alkylated by adding iodoacetamide to a final concentration of 14 mM. Two hundred micrograms of total protein per sample were digested for 16 h at 37 °C using a 50:1 protein:trypsin ratio. For the tandem mass tag (TMT) internal bridge channel, a pooled control containing equal protein amount from all samples was prepared. One-hundred micrograms of protein per sample and the internal control pooled sample were labeled with TMTPro reagents according to manufacturer instructions in a total of two TMTPro plex pooled samples. Multiplexed samples were dried to completion, desalted and basic reverse phase was used to fractionate samples into 12 final concatenated fractions per plex. Samples were analyzed using a Thermo Vanquish Neo (Thermo Scientific) hyphenated to a Thermo Orbitrap Ascend (Thermo Scientific). Approximately 1 μg of protein per fraction was loaded on a trapping column (Thermo Scientific, PepMap100, C18, 300 μm × 5 mm) and resolved on the analytical column (Aurora Ultimate XT 25 cm C18 from IonOpticks) at a flow rate of 300 nl min^−1^ using a gradient of 97% A (0.1% formic acid) 8% B (80% acetonitrile 0.1% formic acid) to 28% B over 90 min, then to 40% B for additional 15 min. Data were acquired using three FAIMS (high-field asymmetric-waveform ion mobility spectrometry) compensation voltages (−40 V, −50 V and −60 V) and each FAIMS experiment had a maximum cycle time of 1.2, 1 and 1 s, respectively. Data-dependent synchronous precursor selection with real-time MS3 trigger (SPS-MS3 RTS) was used for data acquisition and consisted of a 120,000 resolution full-scan MS scan (AGC set to 100% (4 × 10^5^ ions) with a maximum fill time of 50 ms) using a mass range of 400–1,400 *m/z*. To avoid repeated selection of peptides for MS/MS, the program used a 60-s dynamic exclusion window. MS/MS was performed on the ion trap at normal scan rate (AGC set to 200% (2 × 10^4^ ions) with a maximum fill time of 35 ms) with an isolation window of 0.7 *m/z* and an HCD collision energy of 33%. Real-time search parameters were set as follows: *C.* *crescentus* Uniprot database (UP000001364, downloaded in Feb 2025) with trypsin set as the main enzyme, static modifications of cysteine carbamidomethylation (deltaMass = 57.0215) and TMTPro 16plex (deltaMass = 304.2071) on lysines. Methionine oxidation was set as variable modification (deltaMass = 15.99491). One missed cleavage was allowed and false discovery rate (FDR) filtering was enabled. Only five peptides per protein were allowed per basic reverse phase fraction (using the close-out option) and RTS search maximum search time was set to 35 ms. Ten of the most abundant peptide fragments were selected for SPS-MS3 and MS3 acquired at 120,000 resolution on the mass range 100–500 *m/z* with an AGC target of 200% and a maximum injection time of 2,451 ms. MS3 collision energy was set to 55%. Raw data were imported and data were processed in Proteome Discoverer v.3.1 (Thermo Fisher Scientific). The raw files were submitted to a database search using Proteome Discoverer with SequestHF against the *C.* *crescentus* Uniprot database (UP000001364, downloaded in February 2025). Common contaminant proteins (several types of human keratins, BSA and porcine trypsin) were added to the database. The spectra identification was performed with the following parameters: MS accuracy, 10 ppm; MS/MS accuracy of 0.6 Da for spectra acquired in the ion trap mass analyzer; up to two missed cleavage sites allowed; carbamidomethylation of cysteine and TMTPro 16 plex on lysines and peptide N terminus as a fixed modification; and oxidation of methionine as variable modifications. Percolator node was used for FDR estimation and only rank 1 peptide identifications of high confidence (FDR < 1%) were accepted.

Peptide-spectrum match (PSM) level output from Proteome Discoverer was processed in R (v.4.4.1) using QFeatures (v.1.14.2)^62^ and biomasslmb (v.0.0.1) R packages^63^. The R markdown notebooks are available from https://github.com/lmb-mass-spec-compbio/BPAF_uptake_Caulobacter_crescentus_TMT_proteomics and archived by zenodo at 10.5281/zenodo.17659633 (ref. ^64^). PSMs were filtered to remove matches to contaminants and median normalized. PSMs with signal-to-noise ratios <10, co-isolation >50%, peptide matching rank <1 or containing any missing values were removed. Finally, PSMs for proteins with fewer than two PSMs were removed, before summarization to protein level abundances by summing PSM-level intensities. The protein abundances from the two TMT plexes were then combined and normalized using the internal reference standard samples^65^, with proteins present in just one plex removed, leaving 2,986 proteins quantified across all samples. Statistical testing to compare BPAF exposure to control samples at each time point was performed with limma (v.3.60.6)^66^ using the treat function to set the null hypothesis as fold change <1.25 and allowing a trend between mean protein abundance and the prior variance. *P* values were adjusted using the Benjamini–Hochberg FDR procedure^67^ and a threshold of 0.01 (1% FDR) used to identity statistically significant differences. PCA was performed using the prcomp function in R, with center = TRUE. Heatmap visualization of efflux pump proteins was performed using pheatmap (v.1.0.12) with proteins *z*-score normalized across the rows.

### Reporting summary

Further information on research design is available in the Nature Portfolio Reporting Summary linked to this article.

## Online content

Any methods, additional references, Nature Portfolio reporting summaries, source data, extended data, supplementary information, acknowledgements, peer review information; details of author contributions and competing interests; and statements of data and code availability are available at 10.1038/s41592-026-03109-7.

## Supplementary information

Reporting Summary

Peer Review File

Supplementary Table 1 List of detected secondary ionic species. All detected *m/z* secondary ion peaks with an intensity above 10^−6^ counts per extraction are tabulated and identified where possible.

Supplementary Video 1 3D chemical imaging with cryo-FIB-SIMS. Sequential *Z*-stack of the cryo-FIB-SIMS data of the cell shown in Extended Data Fig. 4, illustrating the 3D imaging capability of cryo-FIB-SIMS.

Supplementary Video 2 Tomogram of BPAF-exposed *C.* *crescentus* cell. Tomogram of a *C.* *crescentus* cell exposed to 100 μM BPAF showing the presence of spherical medium-contrast storage granules and darker aggregates.

## Data Availability

All data are available in the main text and the Extended Data materials. Raw FIB-SIMS imaging data files are available upon request. MS proteomics data have been deposited to the ProteomeXchange Consortium via the PRIDE partner repository with the dataset identifier PXD061926.

## References

[1] Beck, M. & Baumeister, W. Cryo-electron tomography: can it reveal the molecular sociology of cells in atomic detail? *Trends Cell Biol.* **26**, 825–837 (2016). 27671779 10.1016/j.tcb.2016.08.006

[2] Henderson, R. The potential and limitations of neutrons, electrons and X-rays for atomic resolution microscopy of unstained biological molecules. *Q. Rev. Biophys.* **28**, 171–193 (1995). 7568675 10.1017/s003358350000305x

[3] Ochner, H. & Bharat, T. A. M. Charting the molecular landscape of the cell. *Structure* **31**, 1297–1305 (2023). 37699393 10.1016/j.str.2023.08.015 PMC7615466

[4] Winograd, N. Imaging mass spectrometry on the nanoscale with cluster ion beams. *Anal. Chem.* **87**, 328–333 (2015). 25458665 10.1021/ac503650p PMC4287836

[5] Fletcher, J. S. Cellular imaging with secondary ion mass spectrometry. *Analyst* **134**, 2204–2215 (2009). 19838405 10.1039/b913575h

[6] Pillatsch, L., Östlund, F. & Michler, J. FIBSIMS: a review of secondary ion mass spectrometry for analytical dual beam focussed ion beam instruments. *Prog. Cryst. Growth Charact. Mater.* **65**, 1–19 (2019).

[7] Stevie, F. A. in *Introduction to Focused Ion Beams* (eds Giannuzzi, L. A. & Stevie, F. A.) 269–280 (Springer, 2005).

[8] McDonnell, L. A. & Heeren, R. M. A. Imaging mass spectrometry. *Mass Spectrom. Rev.* **26**, 606–643 (2007). 17471576 10.1002/mas.20124

[9] Passarelli, M. K. et al. The 3D OrbiSIMS—label-free metabolic imaging with subcellular lateral resolution and high mass-resolving power. *Nat. Methods* **14**, 1175–1183 (2017). 29131162 10.1038/nmeth.4504

[10] Kotowska, A. M. et al. Protein identification by 3D OrbiSIMS to facilitate in situ imaging and depth profiling. *Nat. Commun.* **11**, 5832 (2020). 33203841 10.1038/s41467-020-19445-x PMC7672064

[11] Tian, H., Six, D. A., Krucker, T., Leeds, J. A. & Winograd, N. Subcellular chemical imaging of antibiotics in single bacteria using C_60_ -secondary ion mass spectrometry. *Anal. Chem.* **89**, 5050–5057 (2017). 28332827 10.1021/acs.analchem.7b00466 PMC5415874

[12] Tian, H. et al. Successive high-resolution (H_2_O)_n_-GCIB and C_60_-SIMS imaging integrates multi-omics in different cell types in breast cancer tissue. *Anal. Chem.* **93**, 8143–8151 (2021). 34075742 10.1021/acs.analchem.0c05311 PMC8209780

[13] Zhang, J. et al. Cryo-OrbiSIMS for 3D molecular imaging of a bacterial biofilm in its native state. *Anal. Chem.* **92**, 9008–9015 (2020). 32460495 10.1021/acs.analchem.0c01125

[14] Gilmore, I. S., Heiles, S. & Pieterse, C. L. Metabolic imaging at the single-cell scale: recent advances in mass spectrometry imaging. *Annu. Rev. Anal. Chem.* **12**, 201–224 (2019). 10.1146/annurev-anchem-061318-115516 30848927

[15] De Castro, O. et al. npSCOPE: a new multimodal instrument for in situ correlative analysis of nanoparticles. *Anal. Chem.* **93**, 14417–14424 (2021). 34670088 10.1021/acs.analchem.1c02337

[16] Meibom, A. et al. Correlated cryo-SEM and CryoNanoSIMS imaging of biological tissue. *BMC Biol.* **21**, 126 (2023). 37280616 10.1186/s12915-023-01623-0 PMC10246362

[17] Weber, P. K. et al. The NanoSIMS-HR: the next generation of high spatial resolution dynamic SIMS. *Anal. Chem.* **96**, 19321–19329 (2024). 39591529 10.1021/acs.analchem.4c03091

[18] Lindell, A. E. et al. Human gut bacteria bioaccumulate per- and polyfluoroalkyl substances. *Nat. Microbiol.* **10**, 1630–1647 (2025). 40595288 10.1038/s41564-025-02032-5 PMC12222025

[19] von Kügelgen, A. et al. In situ structure of an intact lipopolysaccharide-bound bacterial surface layer. *Cell* **180**, 348–358.e15 (2020). 31883796 10.1016/j.cell.2019.12.006 PMC6978808

[20] Herdman, M. et al. Cell cycle dependent coordination of surface layer biogenesis in *Caulobacter crescentus*. *Nat. Commun.* **15**, 3355 (2024). 38637514 10.1038/s41467-024-47529-5 PMC11026435

[21] Lork, A. A. et al. Subcellular protein turnover in human neural progenitor cells revealed by correlative electron microscopy and nanoscale secondary ion mass spectrometry imaging. *Chem. Sci.* **15**, 3311–3322 (2024). 38425528 10.1039/d3sc05629e PMC10901485

[22] Rabasco, S. et al. Characterization of stress granule protein turnover in neuronal progenitor cells using correlative STED and NanoSIMS imaging. *Int. J. Mol. Sci.* **24**, 2546 (2023). 36768868 10.3390/ijms24032546 PMC9917160

[23] Bonting, C. F. C., Kortstee, G. J. J., Boekestein, A. & Zehnder, A. J. B. The elemental composition dynamics of large polyphosphate granules in Acinetobacter strain 210A. *Arch. Microbiol.* **159**, 428–434 (1993).

[24] Röske, I., Schönborn, C. & Bauer, H. Influence of the addition of different metals to an activated sludge system on the enhanced biological phosphorus removal. *Int. Rev. Hydrobiol.* **80**, 605–621 (1995).

[25] Tocheva, E. I. et al. Polyphosphate storage during sporulation in the Gram-negative bacterium *Acetonema longum*. *J. Bacteriol.* **195**, 3940–3946 (2013). 23813732 10.1128/JB.00712-13 PMC3754598

[26] De Koning, E. A. et al. The PHB Granule Biogenesis Pathway in *Caulobacter*. Preprint at *bioRxiv*10.1101/2023.07.06.548030 (2023).

[27] Henry, J. T. & Crosson, S. Chromosome replication and segregation govern the biogenesis and inheritance of inorganic polyphosphate granules. *Mol. Biol. Cell* **24**, 3177–3186 (2013). 23985321 10.1091/mbc.E13-04-0182 PMC3806658

[28] Boutte, C. C., Henry, J. T. & Crosson, S. ppGpp and polyphosphate modulate cell cycle progression in *Caulobacter crescentus*. *J. Bacteriol.* **194**, 28–35 (2012). 22020649 10.1128/JB.05932-11 PMC3256613

[29] Comolli, L. R., Kundmann, M. & Downing, K. H. Characterization of intact subcellular bodies in whole bacteria by cryo-electron tomography and spectroscopic imaging. *J. Microsc.* **223**, 40–52 (2006). 16872430 10.1111/j.1365-2818.2006.01597.x

[30] Kukulski, W. et al. Correlated fluorescence and 3D electron microscopy with high sensitivity and spatial precision. *J. Cell Biol.* **192**, 111–119 (2011). 21200030 10.1083/jcb.201009037 PMC3019550

[31] Moser, F. et al. Cryo-SOFI enabling low-dose super-resolution correlative light and electron cryo-microscopy. *Proc. Natl Acad. Sci. USA* **116**, 4804–4809 (2019). 30808803 10.1073/pnas.1810690116 PMC6421404

[32] Herdman, M. et al. High-resolution mapping of metal ions reveals principles of surface layer assembly in *Caulobacter crescentus* cells. *Structure* **30**, 215–228.e5 (2022). 34800371 10.1016/j.str.2021.10.012 PMC8828063

[33] Caspy, I., Wang, Z. & Bharat, T. A. M. Structural biology inside multicellular specimens using electron cryotomography. *Q. Rev. Biophys.* **58**, e6 (2025). 39801355 10.1017/S0033583525000010 PMC7617309

[34] Marko, M., Hsieh, C., Schalek, R., Frank, J. & Mannella, C. Focused-ion-beam thinning of frozen-hydrated biological specimens for cryo-electron microscopy. *Nat. Methods* **4**, 215–217 (2007). 17277781 10.1038/nmeth1014

[35] Heymann, J. A. W. et al. Site-specific 3D imaging of cells and tissues with a dual beam microscope. *J. Struct. Biol.* **155**, 63–73 (2006). 16713294 10.1016/j.jsb.2006.03.006 PMC1647295

[36] Baumeister, W. Electron tomography: towards visualizing the molecular organization of the cytoplasm. *Curr. Opin. Struct. Biol.* **12**, 679–684 (2002). 12464323 10.1016/s0959-440x(02)00378-0

[37] Escrivá, L., Hanberg, A., Zilliacus, J. & Beronius, A. Assessment of the endocrine disrupting properties of bisphenol AF according to the EU criteria and ECHA/EFSA guidance. *EFS2*10.2903/j.efsa.2019.e170914 (2019). 10.2903/j.efsa.2019.e170914 PMC7015508 32626472

[38] Kahil, K. et al. Elemental compositions of sea urchin larval cell vesicles evaluated by cryo-STEM-EDS and cryo-SEM-EDS. *Acta Biomater.* **155**, 482–490 (2023). 36375785 10.1016/j.actbio.2022.11.012

[39] Toso, D. B., Henstra, A. M., Gunsalus, R. P. & Zhou, Z. H. Structural, mass and elemental analyses of storage granules in methanogenic archaeal cells. *Env. Microbiol.* **13**, 2587–2599 (2011). 21854518 10.1111/j.1462-2920.2011.02531.x PMC3700383

[40] Toso, D. B., Javed, M. M., Czornyj, E., Gunsalus, R. P. & Zhou, Z. H. Discovery and characterization of iron sulfide and polyphosphate bodies coexisting in *Archaeoglobus fulgidus* cells. *Archaea* **2016**, 4706532 (2016). 27194953 10.1155/2016/4706532 PMC4853940

[41] Elad, N., Bellapadrona, G., Houben, L., Sagi, I. & Elbaum, M. Detection of isolated protein-bound metal ions by single-particle cryo-STEM. *Proc. Natl Acad. Sci. USA* **114**, 11139–11144 (2017). 28973937 10.1073/pnas.1708609114 PMC5651765

[42] Wolf, S. G., Houben, L. & Elbaum, M. Cryo-scanning transmission electron tomography of vitrified cells. *Nat. Methods* **11**, 423–428 (2014). 24531421 10.1038/nmeth.2842

[43] Aronova, M. A. & Leapman, R. D. Development of electron energy-loss spectroscopy in the biological sciences. *MRS Bull*. **37**, 53–62 (2012). 23049161 10.1557/mrs.2011.329 PMC3465455

[44] Priebe, A. & Michler, J. Review of recent advances in gas-assisted focused ion beam time-of-flight secondary ion mass spectrometry (FIB-TOF-SIMS). *Materials* **16**, 2090 (2023). 36903205 10.3390/ma16052090 PMC10003971

[45] Ramakrishna, P. et al. Elemental cryo-imaging reveals SOS1-dependent vacuolar sodium accumulation. *Nature* **637**, 1228–1233 (2025). 39814877 10.1038/s41586-024-08403-y PMC11779634

[46] Lange, F. et al. Correlative fluorescence microscopy, transmission electron microscopy and secondary ion mass spectrometry (CLEM-SIMS) for cellular imaging. *PLoS ONE* **16**, e0240768 (2021). 33970908 10.1371/journal.pone.0240768 PMC8109779

[47] Winograd, N. The magic of cluster SIMS. *Anal. Chem.* **77**, 142 A–149 A (2005).

[48] Esser, T. K. et al. Cryo-EM of soft-landed β-galactosidase: gas-phase and native structures are remarkably similar. *Sci. Adv.* **10**, eadl4628 (2024). 38354247 10.1126/sciadv.adl4628 PMC10866560

[49] O’Reilly, F. J. et al. In-cell architecture of an actively transcribing-translating expressome. *Science* **369**, 554–557 (2020). 32732422 10.1126/science.abb3758 PMC7115962

[50] Pfeil-Gardiner, O. et al. Elemental mapping in single-particle reconstructions by reconstructed electron energy-loss analysis. *Nat. Methods*10.1038/s41592-024-02482-5 (2024). 10.1038/s41592-024-02482-5 PMC11621030 39448878

[51] Schwarz, A. et al. Correlative MS imaging for in situ cryo-ET. Preprint at *bioRxiv*10.1101/2025.09.16.676641 (2025).

[52] Bharat, T. A. M. et al. Structure of the hexagonal surface layer on *Caulobacter crescentus* cells. *Nat. Microbiol.* **2**, 17059 (2017). 28418382 10.1038/nmicrobiol.2017.59 PMC5699643

[53] Sulkowski, N. I., Hardy, G. G., Brun, Y. V. & Bharat, T. A. M. A multiprotein complex anchors adhesive holdfast at the outer membrane of *Caulobacter crescentus*. *J. Bacteriol.* **201**, e00112–e00119 (2019). 31061167 10.1128/JB.00112-19 PMC6707917

[54] Isbilir, B., Yeates, A., Alva, V. & Bharat, T. A. M. Mapping the ultrastructural topology of the corynebacterial cell surface. *PLoS Biol.* **23**, e3003130 (2025). 10.1371/journal.pbio.3003130 PMC12021427 40233127

[55] Kelley, K. et al. Waffle method: a general and flexible approach for improving throughput in FIB-milling. *Nat. Commun.* **13**, 1857 (2022). 35387991 10.1038/s41467-022-29501-3 PMC8987090

[56] Fu, J., Joshi, S. B. & Catchmark, J. M. Sputtering rate of micromilling on water ice with focused ion beam in a cryogenic environment. *J. Vac. Sci. Technol.* **26**, 422–429 (2008).

[57] Mastronarde, D. N. Automated electron microscope tomography using robust prediction of specimen movements. *J. Struct. Biol.* **152**, 36–51 (2005). 16182563 10.1016/j.jsb.2005.07.007

[58] Lowe, D. G. Distinctive image features from scale-invariant keypoints. *Int. J. Comput. Vis.* **60**, 91–110 (2004).

[59] Schindelin, J. et al. Fiji: an open-source platform for biological-image analysis. *Nat. Methods* **9**, 676–682 (2012). 22743772 10.1038/nmeth.2019 PMC3855844

[60] Zheng, S. et al. AreTomo: an integrated software package for automated marker-free, motion-corrected cryo-electron tomographic alignment and reconstruction. *J. Struct. Biol. X* **6**, 100068 (2022). 35601683 10.1016/j.yjsbx.2022.100068 PMC9117686

[61] Kremer, J. R., Mastronarde, D. N. & McIntosh, J. R. Computer visualization of three-dimensional image data using IMOD. *J. Struct. Biol.* **116**, 71–76 (1996). 8742726 10.1006/jsbi.1996.0013

[62] Gatto, L. et al. QFeatures: quantitative features for mass spectrometry data. R package v.1.22.0 10.18129/B9.BIOC.QFEATURES (2026).

[63] Smith, T. biomasslmb: BioMass LMB proteomics data analysis utility functions. R package v.0.0.1 https://lmb-mass-spec-compbio.github.io/biomasslmb/ (2025).

[64] Smith, T. lmb-mass-spec-compbio/BPAF_uptake_*Caulobacter_crescentus*_TMT_proteomics: manuscript submission. *Zenodo*10.5281/zenodo.17659633 (2025).

[65] Plubell, D. L. et al. Extended multiplexing of tandem mass tags (TMT) labeling reveals age and high fat diet specific proteome changes in mouse epididymal adipose tissue. *Mol. Cell. Proteom.* **16**, 873–890 (2017). 10.1074/mcp.M116.065524 PMC5417827 28325852

[66] Ritchie, M. E. et al. limma powers differential expression analyses for RNA-sequencing and microarray studies. *Nucleic Acids Res.* **43**, e47 (2015). 25605792 10.1093/nar/gkv007 PMC4402510

[67] Benjamini, Y. & Hochberg, Y. Controlling the false discovery rate: a practical and powerful approach to multiple testing. *J. R. Stat. Soc. B* **57**, 289–300 (1995).

