# Supplementary material for: Subcellular chemical mapping using correlated cryogenic electron and mass spectrometry imaging
Source: Nat Methods. 2026 May 25;23(6):1174–83. doi: 10.1038/s41592-026-03109-7 (PMC13259928; doi:10.1038/s41592-026-03109-7)
Supplement: Supplementary file 3 — List of detected secondary ionic species. All detected m/z secondary ion peaks with an intensity above 10−6 counts per extraction are tabulated and identified where possible. [file 41592_2026_3109_MOESM3_ESM.pdf]

Ions detected in negative mode, peaks with intensities > 10^{-6} counts/extraction

12	C
13	^{13}C, CH
16	O
17	OH
18	?
19	F
24	C2
25	C_2H
26	CN
27	CHN, ^{13}CN
31	P
32	O_2, S?
33	HO_2? HS?
34	H_2O_2?
35	Cl
36	C_3
37	Cl isotope
38	C_2N, F_2?
39	C_2HN?
40	CN_2?
41	CHN_2
42	NCO, C_2H_2O, C_2H_4N
43	C_2H_3O, CHNO
45	HCO_2, C_2H_5O
47	PO, CH_3S
48	C_4
49	C_4H
50	C_3N
51	C_3HN?
59	$C_2H_3O_2$
60	C_5
61	HN_2O_2
62	NO_3
63	PO_2
64	SO_2
65	S_2H, C_3HN_2
66	C_3NO, C_4H_4N
69	CF_3?
72	C_6, $C_2H_2NO_2$
73	C_6H, $C_2H_3NO_2$

74	$C_2H_4NO_2$, C_2H_4NS
76	CO_4?
77	$C_2H_5O_3$
79	PO_3
84	C_7
85	$C_3H_3NO_2$, $C_4H_5O_2$
87	$C_3H_3O_3$
93	C_6H_5O, $C_5H_5N_2$
95	PO_4
96	C_8
97	C_8H
98	$C_4H_4NO_2$
101	GaO_2?
103	GaO_2H_2?, $C_3H_3O_4$?
108	C_6H_6NO, C_9
109	C_9H
117	$C_4H_5O_4$?
119	C_8H_7O?
120	C_{10}
121	?
132	C_{11}
133	$C_4H_7NO_4$ (aspartic acid)?
135	$C_5H_5N_5$ (adenine)?
144	C_{12}
145	glutamine or lysine?
156	C_{13}
168	C_{14}
194	Pt isotope
195	Pt isotope
196	Pt isotope
198	Pt isotope

Ions detected in positive mode, peaks with intensities > 10^{-6} counts/extraction

12	C
13	CH
14	N, CH_2
16	O, NH_2
17	NH_3, OH?
18	H_2O, NH_4
19	H_3O
20	?
23	Na
24	Mg
25	Mg isotope? C_2H?
26	Mg isotope? C_2H_2? CN?
27	CNH
28	CH_2N, CO?
29	C_2H_5, CHO?
30	CH_4N
37	H_3OH_2O
39	K, C_3H_3
40	Ca
41	C_3H_5
42	C_2H_4N
43	C_2H_5N
44	C_2H_6N
45	C_2H_7N, C_2H_5O
47	C_2H_7O
55	$H_3O(H_2O)_2$, C_3H_3O
56	Fe, C_3H_6N
57	C_3H_5O, C_3H_7N
58	C_2H_4NO
59	C_3H_7O
60	C_2H_6NO
61	$C_2H_5O_2$
63	Cu?
65	? Cu isotope?
69	Ga
71	Ga
72	C_3H_6NO

73	$H_3O(H_2O)_3$, $C_3H_5O_2$?
75	$C_2H_5NO_2$
77	C_6H_5
81	$C_4H_5N_2$
83	$C_4H_7N_2$
85	?
86	C_4H_8NO, $C_5H_{12}N$
87	C_4H_9NO, $C_5H_{11}O$, $C_5N_{13}N$
88	$C_3H_6NO_2$
89	C_7H_5, $C_3H_9N_2O$
91	$H_3O(H_2O)_4$
93	$C_5H_5N_2$
95	$C_5H_7N_2$
96	$C_6H_{10}N$
99	$C_5H_7O_2$?
103	C_8H_7, $C_4H_{11}N_2O$
104	$C_4H_{10}NS$
105	$C_3H_5O_2S$, C_8H_9
106	$C_3H_8NO_3$
107	C_7H_7O, $C_3H_9NO_3$
109	$H_3O(H_2O)_5$, $C_6H_5O_2$?
111	$C_6H_7O_2$?
113	$C_5H_5O_3$?
121	C_8H_9O
122	?
123	?
125	$C_6H_9O_3$?
127	$H_3O(H_2O)_6$
138	Ga_2
140	Ga_2
142	Ga_2
145	$H_3O(H_2O)_7$
154	?
155	?
156	?
157	?
163	$H_3O(H_2O)_8$
181	$H_3O(H_2O)_9$
195 (193-197)	Pt
199	$H_3O(H_2O)_{10}$

Ions marked in blue are known amino acid fragments[1,2].

Ions marked in red are known monosaccharide fragments[3,4].

Ions marked in green are water clusters.

Ions marked in orange are related to ^{13}C-labelling and thus occur in the corresponding samples.

Ions marked in pink are BPAF fragments and occur in the corresponding samples.

There are several detectable peaks above 200 m/z, both in positive and negative mode. The clustering of these peaks suggests that they are mainly related to the platinum-based compound used for sample backside coating.

1. Kawecki, M. & Bernard, L. Database of proteinogenic amino acid reference spectra for Bismuth-cluster ToF-SIMS. II. Positive polarity. *Surface Science Spectra* **25**, 015002 (2018).
2. Kawecki, M. & Bernard, L. Database of proteinogenic amino acid reference spectra for Bismuth-cluster ToF-SIMS. I. Negative polarity. *Surface Science Spectra* **25**, 015001 (2018).
3. Bernard, L., Crockett, R. & Kawecki, M. Monosaccharides: A ToF-SIMS reference spectra database. II. Positive polarity. *Surface Science Spectra* **26**, 025002 (2019).
4. Bernard, L., Crockett, R. & Kawecki, M. Monosaccharides: A ToF-SIMS reference spectra database. I. Negative polarity. *Surface Science Spectra* **26**, 025001 (2019).
