# Supplementary material for: Subcellular chemical mapping using correlated cryogenic electron and mass spectrometry imaging
Source: Nat Methods. 2026 May 25;23(6):1174–83. doi: 10.1038/s41592-026-03109-7 (PMC13259928; doi:10.1038/s41592-026-03109-7)
Supplement: Supplementary file 2 — Peer Review File [file 41592_2026_3109_MOESM2_ESM.pdf]

nature portfolio

Peer Review File

Sub-cellular chemical mapping using correlated cryogenic electron and mass spectrometry imaging

Corresponding Author: Dr Tanmay Bharat

This file contains all editorial decision letters in order by version, followed by all author rebuttals in order by version.

Version 0:

Decision Letter:

26th Sep 2025

Dear Dr. Bharat,

Thank you for transferring your Article, "Sub-cellular chemical mapping in bacteria using correlated cryogenic electron and mass spectrometry imaging" to Nature Methods. We have evaluated your revision plan and rebuttal letter and we invite you to submit a revised manuscript, as you have outlined.

When revising your paper:

* include a point-by-point response to the reviewers and to any editorial suggestions

* please underline/highlight any additions to the text or areas with other significant changes to facilitate review of the revised manuscript

* address the points listed described below to conform to our open science requirements

* ensure it complies with our general format requirements as set out in our guide to authors at www.nature.com/naturemethods

* resubmit all the necessary files by using the link below to access your home page

Link Redacted

Note: This URL links to your confidential account and associated information about manuscripts you may have submitted, or that you are reviewing for us. If you wish to forward this email to co-authors, please delete this link.

We hope to receive your revised paper within 2 months. If you are substantially delayed, please let us know. In this event, we will still be happy to reconsider your paper at a later date so long as nothing similar has been accepted for publication at Nature Methods or published elsewhere.

OPEN SCIENCE REQUIREMENTS

REPORTING SUMMARY
When revising your manuscript, please update your reporting summary.

Reporting summary: https://www.nature.com/documents/nr-reporting-summary.zip

If your paper includes custom software, we also ask you to complete a supplemental reporting summary.

Software supplement: https://www.nature.com/documents/nr-software-policy.pdf

Please submit these with your revised manuscript. They will be available to reviewers to aid in their evaluation if the paper is re-reviewed. If you have any questions about the checklist, please see http://www.nature.com/authors/policies/availability.html or contact me.

Please note that these forms are dynamic 'smart pdfs' and must therefore be downloaded and completed in Adobe Reader. We will then flatten them for ease of use by the reviewers. If you would like to reference the guidance text as you complete the template, please access these flattened versions at http://www.nature.com/authors/policies/availability.html.

For any revision that includes light microscopy data, we ask our authors to please include a completed light microscopy reporting table [https://www.nature.com/documents/Light_microscopy_reporting_table.xlsx] to ensure the methods are described thoroughly. The table will be available to reviewers and ultimately published should the manuscript be accepted at the journal.

EXTENDED DATA FIGURES
When re-submitting your manuscript, please ensure that any supplementary figures and tables that are crucial to the manuscript's conclusions are converted into Extended Data figures and tables to increase visibility of these data. Extended Data figures and tables are online-only (present in the online PDF and full-text HTML versions of the paper), peer-reviewed display items that provide essential background to the article but are not included in the main article due to space constraints. A maximum of ten Extended Data display items (figures and tables) is permitted.

DATA AVAILABILITY
We strongly encourage you to deposit all new data associated with the paper in a persistent repository where they can be freely and enduringly accessed. We recommend submitting the data to discipline-specific and community-recognized repositories; a list of repositories is provided here: http://www.nature.com/sdata/policies/repositories

All novel DNA and RNA sequencing data, protein sequences, genetic polymorphisms, linked genotype and phenotype data, gene expression data, macromolecular structures, and proteomics data must be deposited in a publicly accessible database, and accession codes and associated hyperlinks must be provided in the "Data Availability" section.

For papers containing bioimaging data, we strongly recommend depositing the data to Bioimage Archive (https://www.ebi.ac.uk/bioimage-archive/). Associated accession codes and hyperlinks should be provided in the "Data Availability" section.

Refer to our data policies here: https://www.nature.com/nature-research/editorial-policies/reporting-standards#availability-of-data

To further increase transparency, we encourage you to provide, in tabular form, the data underlying the graphical representations used in your figures. This is in addition to our data-deposition policy for specific types of experiments and large datasets. For readers, the source data will be made accessible directly from the figure legend. Spreadsheets can be submitted in .xls, .xlsx or .csv formats. Only one (1) file per figure is permitted: thus if there is a multi-paneled figure the source data for each panel should be clearly labeled in the csv/Excel file; alternately the data for a figure can be included in multiple, clearly labeled sheets in an Excel file. File sizes of up to 30 MB are permitted. When submitting source data files with your manuscript please select the Source Data file type and use the Title field in the File Description tab to indicate which figure the source data pertains to.

Please include a "Data availability" subsection in the Online Methods. This section should inform readers about the availability of the data used to support the conclusions of your study, including accession codes to public repositories, references to source data that may be published alongside the paper, unique identifiers such as URLs to data repository entries, or data set DOIs, and any other statement about data availability. At a minimum, you should include the following statement: "The data that support the findings of this study are available from the corresponding author upon request", describing which data is available upon request and mentioning any restrictions on availability. If DOIs are provided, please include these in the Reference list (authors, title, publisher (repository name), identifier, year). For more guidance on how to write this section please see: http://www.nature.com/authors/policies/data/data-availability-statements-data-citations.pdf

MATERIALS AVAILABILITY
As a condition of publication in Nature Methods, authors are required to make unique materials promptly available to others without undue qualifications.

Authors reporting new chemical compounds must provide chemical structure, synthesis and characterization details. Authors reporting mutant strains and cell lines are strongly encouraged to use established public repositories.

More details about our materials availability policy can be found at https://www.nature.com/nature-portfolio/editorial-policies/reporting-standards#availability-of-materials

SUPPLEMENTARY PROTOCOL

To help facilitate reproducibility and uptake of your method, we ask you to prepare a step-by-step Supplementary Protocol for the method described in this paper. We encourage authors to share their step-by-step experimental protocols on a protocol sharing platform of their choice and report the protocol DOI in the reference list. Nature Portfolio's protocols.io is a free-to-use and open resource for protocols; protocols deposited onto protocols.io are citable and can be linked from the published article. More details can found at protocols.io.

ORCID
Nature Methods is committed to improving transparency in authorship. As part of our efforts in this direction, we are now requesting that all authors identified as 'corresponding author' on published papers create and link their Open Researcher and Contributor Identifier (ORCID) with their account on the Manuscript Tracking System (MTS), prior to acceptance. This applies to primary research papers only. ORCID helps the scientific community achieve unambiguous attribution of all scholarly contributions. You can create and link your ORCID from the home page of the MTS by clicking on 'Modify my Springer Nature account'. For more information please visit please visit www.springernature.com/orcid.

Please do not hesitate to contact me if you have any questions or would like to discuss these revisions further. We look forward to seeing your revised manuscript and thank you for the opportunity to consider your work.

Best regards,
Allison

Allison Doerr, Ph.D.
Chief Editor
Nature Methods

Version 1:

Decision Letter:

Our ref: NMETH-A62612A

14th Jan 2026

Dear Tanmay,

Thank you for submitting your revised manuscript "Sub-cellular chemical mapping using correlated cryogenic electron and mass spectrometry imaging" (NMETH-A62612A). It has now been seen by the original referees and their comments are below. The reviewers find that the paper has improved in revision, and therefore we'll be happy in principle to publish it in Nature Methods, pending minor revisions to satisfy the referees' final requests and to comply with our editorial and formatting guidelines.

We are now performing detailed checks on your paper and will send you a checklist detailing our editorial and formatting requirements within two weeks or so. Please do not upload the final materials and make any revisions until you receive this additional information from us.

TRANSPARENT PEER REVIEW
Nature Methods offers a transparent peer review option for new original research manuscripts submitted from 17th February 2021. We encourage increased transparency in peer review by publishing the reviewer comments, author rebuttal letters and editorial decision letters if the authors agree. Such peer review material is made available as a supplementary peer review file. **Please state in the cover letter 'I wish to participate in transparent peer review' if you want to opt in, or 'I do not wish to participate in transparent peer review' if you don't.** Failure to state your preference will result in delays in accepting your manuscript for publication.

Please note: we allow redactions to authors' rebuttal and reviewer comments in the interest of confidentiality. If you are concerned about the release of confidential data, please let us know specifically what information you would like to have removed. Please note that we cannot incorporate redactions for any other reasons. Reviewer names will be published in the peer review files if the reviewer signed the comments to authors, or if reviewers explicitly agree to release their name. For more information, please refer to our FAQ page.

ORCID
IMPORTANT: Non-corresponding authors do not have to link their ORCIDs but are encouraged to do so. Please note that it will not be possible to add/modify ORCIDs at proof. Thus, please let your co-authors know that if they wish to have their ORCID added to the paper they must follow the procedure described in the following link prior to acceptance:
https://www.springernature.com/gp/researchers/orcid/orcid-for-nature-research

Author names using non-Roman characters

Nature Portfolio journals can support presentation of author names using non-Roman characters in the HTML version of the article. If you wish to, please include author names in parentheses after the Roman-character spelling; see example online here. Currently supported scripts are: Arabic, Chinese, Cyrillic, Devanagari, Greek, Hebrew, Hangul, Japanese and Persian. You will be asked to verify the rendering is correct at proof stage.

Thank you again for your interest in Nature Methods. Please do not hesitate to contact me if you have any questions. We will be in touch again soon.

Sincerely yours,
Allison

Allison Doerr, Ph.D.
Chief Editor
Nature Methods

Reviewer #1 (Remarks to the Author):

In the resubmitted manuscript by Ochner et al., the authors have added substantial new data and strengthened the discussion and description of their workflow, which significantly improves their work. They have thoughtfully addressed the major and minor points raised by the reviewers. In particular, the manuscript now much more clearly conveys how the approach is distinct from -and complementary to - other chemical analytical tools used alongside cryo-ET and cryo-EM, supported by convincing new results highlighting isotope detection. The revised methodological description is also clearer overall, and the annotated photographs of the instrumental setup are especially helpful.

The authors also include yeast lamellae as a eukaryotic proof-of-principle example, demonstrating differential ion distributions in a more complex cytosolic environment. Given that the authors report analyzing ~20 lamellae, but only show two examples, it would be wonderful if they could add a brief summary of what they learned from this dataset; for example, whether any consistent trends emerged across lamellae, and whether particular distribution patterns could be linked to organelles identifiable in the corresponding cryo-ET/cryo-EM data. As one example, the K abundance and distribution appear different between Figure 3f and Supplementary Figure 7i; it would be helpful to know whether the authors think this reflects biological variability, different membrane compartments, differences in lamella/sample preparation, or other experimental factors. If drawing broader conclusions from the current yeast dataset is challenging without additional targeted experiments, perhaps the authors could comment instead.

Overall, the presented cryo-FIB-SIMS method and the correlative cryo-EM/cryo-ET workflow represent an exciting new spatio-chemical imaging approach and a valuable addition to the correlative cryo-EM and cryo-ET toolkit and I am looking forward to its publication. The remaining point for me is minor and relates only to providing a bit more context on the yeast lamella results, if feasible. The revised manuscript is very well and clearly written.

Reviewer #3 (Remarks to the Author):

Hannah Ochner and colleagues thoroughly revised their exellently-written manuscript titled

"Sub-cellular chemical mapping using correlated cryogenic electron and mass spectrometry imaging"

on a novel correlative workflow to analyze structure and chemical composition of bacteria at sub-cellular level.
In their revised manuscript the authors included the missing references to important publications in the field of correlative microscopy of biological specimens under cryogenic conditions and discussed how their work is complementary to these studies. The authors modified parts of the discussion and pointed out how ToF-SIMS, being the central analytical technique in their workflow, allows for localizing molecules of interest on sub-cellular or, in case of eukaryotic cells, sub-organellar level. In my first review I challenged the authors with the question why they are using a ToF-SIMS instead of an energy-dispersive X-ray spectrometer as their major application was the identification of chemical elements in the sample. They correctly argued that SIMS has the advantage to provide information about molecules ad isotopes and proved it by publishing the list of detected ions in negative extraction mode. As a proof-of-principle an additional experiment with 13C labelled cells was conducted which I find very convincing.

Overall, I think this is an excellent piece of work that in my opinion is now ready for publication in Nature Methods.

Version 2:

Decision Letter:

24th Apr 2026

Dear Tanmay,

I am pleased to inform you that your Article, "Sub-cellular chemical mapping using correlated cryogenic electron and mass spectrometry imaging", has now been accepted for publication in Nature Methods. The received and accepted dates will be 3 September 2025 and 24 April 2026. This note is intended to let you know what to expect from us over the next month or so, and to let you know where to address any further questions.

Over the next few weeks, your paper will be copyedited to ensure that it conforms to Nature Methods style. Once your paper is typeset, you will receive an email with a link to choose the appropriate publishing options for your paper and our Author Services team will be in touch regarding any additional information that may be required. It is extremely important that you let us know now whether you will be difficult to contact over the next month. If this is the case, we ask that you send us the contact information (email, phone and fax) of someone who will be able to check the proofs and deal with any last-minute problems.

After the grant of rights is completed, you will receive a link to your electronic proof via email with a request to make any corrections within 48 hours. If, when you receive your proof, you cannot meet this deadline, please inform us at rjsproduction@springernature.com immediately.

Authors may need to take specific actions to achieve compliance with funder and institutional open access mandates. If your research is supported by a funder that requires immediate open access (e.g. according to Plan S principles or the NIH public access policy) then you should select the gold OA route, and we will direct you to the compliant route where possible. Because authors warrant under our subscription licensing terms that they haven’t committed to licensing any version of their article under a licence inconsistent with the terms of our agreement – including the applicable embargo period – publication under the subscription model isn’t suitable for authors whose funders require no embargo.

If you have any questions about our publishing options, costs, Open Access requirements, or our legal forms, please contact ASJournals@springernature.com

If you have posted a preprint on any preprint server, please ensure that the preprint details are updated with a publication reference, including the DOI and a URL to the published version of the article on the journal website.

You may wish to make your media relations office aware of your accepted publication, in case they consider it appropriate to organize some internal or external publicity. Once your paper has been scheduled you will receive an email confirming the publication details. This is normally 3-4 working days in advance of publication. If you need additional notice of the date and time of publication, please let the production team know when you receive the proof of your article to ensure there is sufficient time to coordinate. Further information on our embargo policies can be found here: https://www.nature.com/authors/policies/embargo.html

To assist our authors in disseminating their research to the broader community, our SharedIt initiative provides you with a unique shareable link that will allow anyone (with or without a subscription) to read the published article. Recipients of the link with a subscription will also be able to download and print the PDF.

As soon as your article is published, you will receive an automated email with your shareable link.

If you are active on Twitter/X or Bluesky, please e-mail me your and your coauthors’ handles so that we may tag you when the paper is published.

You can now use a single sign-on for all your accounts, view the status of all your manuscript submissions and reviews, access usage statistics for your published articles and download a record of your refereeing activity for the Nature journals.

Please note that you and any of your coauthors will be able to order reprints and single copies of the issue containing your article through Nature Portfolio's reprint website, which is located at http://www.nature.com/reprints/author-reprints.html. If there are any questions about reprints please send an email to author-reprints@nature.com and someone will assist you.

Please feel free to contact me if you have questions about any of these points.

Best regards,
Allison

Allison Doerr, Ph.D.
Chief Editor
Nature Methods

** Visit the Springer Nature Editorial and Publishing website at www.springernature.com/editorial-

and-publishing-jobs for more information about our career opportunities. If you have any questions please click here.**

Open Access This Peer Review File is licensed under a Creative Commons Attribution 4.0 International License, which permits use, sharing, adaptation, distribution and reproduction in any medium or format, as long as you give appropriate credit to the original author(s) and the source, provide a link to the Creative Commons license, and indicate if changes were made. In cases where reviewers are anonymous, credit should be given to 'Anonymous Referee' and the source.
The images or other third party material in this Peer Review File are included in the article's Creative Commons license, unless indicated otherwise in a credit line to the material. If material is not included in the article's Creative Commons license and your intended use is not permitted by statutory regulation or exceeds the permitted use, you will need to obtain permission directly from the copyright holder.
To view a copy of this license, visit https://creativecommons.org/licenses/by/4.0/

<u>Response to reviewers' comments for manuscript NMETH-A62612-T, Ochner *et al.*</u>

We thank the reviewers for their time and critical comments, which have helped us improve our manuscript. We have addressed the two main points of concern, i.e. a demonstration of the generalizability of our method to eukaryotic samples and the comparison of our technique to similar workflows.

1) To show that our workflow can be generalized to eukaryotic samples, we have updated Fig. 3 and Extended Data Figure 7 to include data from lamellae of plunge-frozen yeast cells (Fig. 3f and Extended Data Figure 7i) alongside the bacterial example already presented.

2) To position our technique more clearly within the field of spatially resolved chemical imaging, we provide a systematic comparison of our method to other techniques such as EELS/EDX as well as SIMS-based methods, such as correlative nanoSIMS, specifically regarding the imaging of biological specimens under cryogenic conditions.

    a. To this end, we provide a supplementary table enumerating the ions identified in our SIMS data (Supplementary Table S1). As reviewer 3 points out, a large subset of these, namely molecular ions, cannot be detected in other techniques such as EELS/EDX, which rely on elemental atomic characteristics. This enumeration shows the complementarity of our workflow with EELS/EDX.
    b. We also provide new experimental data showing the detection of different isotopes of the same element (Extended Data Figure 5c), which are not detectable in EELS/EDX.
    c. In addition, we provide an in-depth discussion about how our workflow is complementary to other chemical imaging methods.
    d. Finally, we have also extended the discussion to highlight future possibilities for the detection of larger molecules. Larger molecules, including whole drug molecules and peptides, could be detected by adapting our workflow to include the use of larger primary ion beams, which are regularly employed in other types of SIMS experiments.

Specific point-by-point responses to the reviewers' comments are below.

Referee expertise:

Referee #1: cryo-EM/ET

Referee #2: EM technology development

Referee #3: SIMS, biological sample preparation, FIB and SEM

Referees' comments:

Referee #1 (Remarks to the Author):

The study by Ochner et al. presents a new workflow that combines high resolution spatial imaging by cryoEM with spatial chemical identity analysis through cryo FIB secondary ion mass spectrometry (SIMS) in a correlative manner. The authors first demonstrate the cryo FIB SIMS method on standard testing samples. Subsequently the authors demonstrate that cryo FIB SIMS is capable of detecting gold labeling at the surface of bacteria, works on bacteria lamella and in correlation with cryo fluorescence microscopy. Ultimately, the authors use the presented workflow to study the uptake of BPAF, a contaminant from plastics production, by bacteria. The authors first demonstrate the effect from BPAF uptake on bacteria growth, the bacterial proteome and observe an increased expression of efflux pump related proteins. Finally, using correlative cryoEM and cryo FIB-SIMS imaging the authors locate the presence of fluorine - from BPAF - to storage granules.

The development of methods combining the high spatial resolution in native frozen samples in cryoEM with techniques that determine the chemical identity within subregions of the sample is an exciting topic for the cryoEM and cryoET field. Ultimately the field is waiting for technology that allows the identification of proteins through peptides but as discussed by the authors this is not yet possible and requires more development of the technology. The presented workflow has the capability to identify different ions, metals, certain molecules and isotopes in the sample and allows correlating those to specific regions inside the EM or ET data which has the potential to encourage new cryoEM/ET project avenues.

While a cryo-nano-SIMS instrument for correlative cryo SEM work (Meibom, A. et al. BMC Biol 21, 126 (2023))

was previously presented, the instrumental set up and sample preparation is different and the authors have focused on integrating the cryoSIMS application into the existing cryoET and FIB milling workflows and instruments which is beneficial for the cryoEM/cryoET field. Since one main novelty is the workflow, the manuscript would benefit from additional details on the set up of the instrument, the workflow optimization as well as a more extended comparison to other workflows that can provide similar types of data (cryo nano SIMS, EELS, EDX) - for instance in previous works others have used cryoEM/ET together with STEM EDX to characterize the chemical composition of storage granules in archaea (Toso, D. et al. Environmental Microbiology 2011, Toso, D. et al., Archaea, 2016).

To provide more information on instrument setup and workflow, we have added a photograph of the outside and inside of the instrument as a new Extended Data Figure 2, and expanded the description of the instrument and workflow in the main text and the methods section.

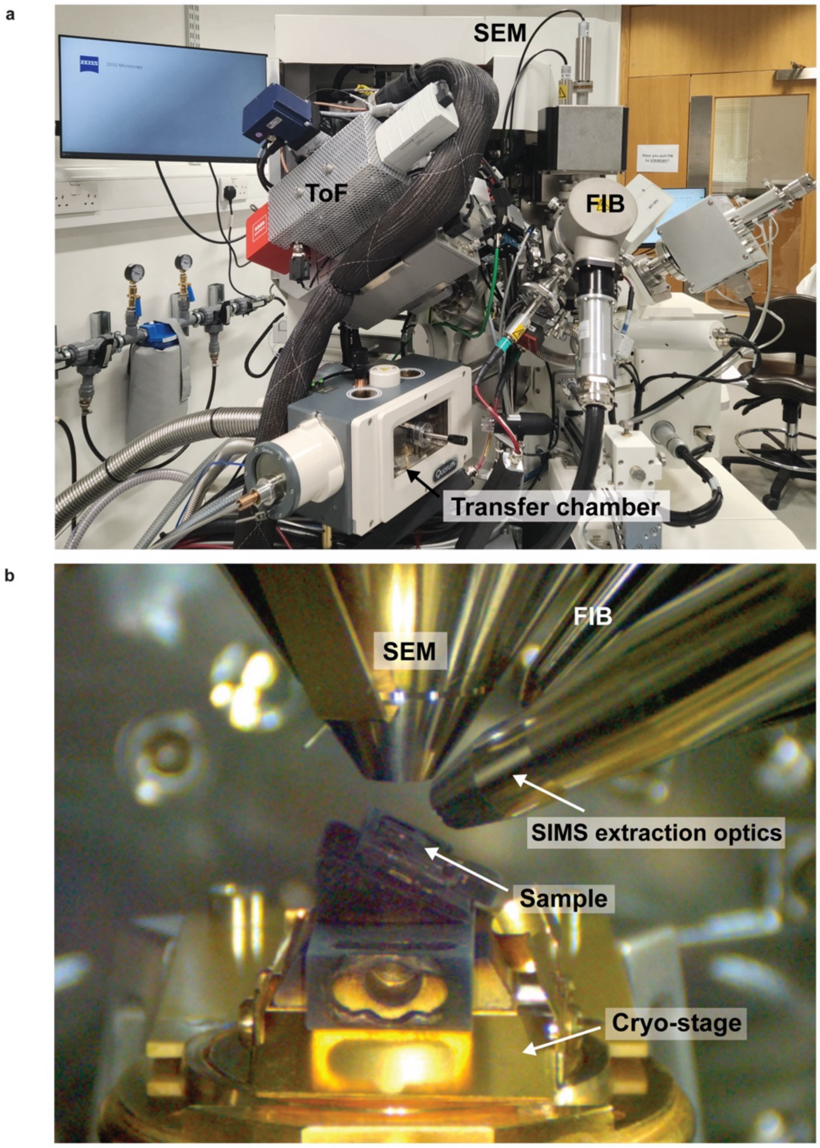

Extended Data Fig. 2: Cryo-FIB-SIMS instrument setup. a Photograph of the outside of the machine with relevant components marked. **b** Photograph of the inside of the microscope chamber with SIMS extraction optics inserted. Relevant components have been highlighted.

Line 83-88:
"To perform SIMS imaging, a focused gallium (Ga) ion beam oriented perpendicular to the sample plane (Extended Data Figure 2b), with an unbinned pixel size range in MS imaging mode of approximately 10-30 nm, is used to scan the field of view, ablating material from the specimen (Extended Data Figure 1b). The secondary ions produced by this ablation are extracted into the ToF mass spectrometer (at nearly the same angular orientation as the FIB) at every pixel of the scan, yielding a mass spectrum for each pixel."

Line 675-683:

"The imaging geometry is of the cryo-FIB-SIMS instrument is depicted in Extended Data Figure 2. The FIB column and the ToF mass spectrometer have nearly the same angular orientation with respect to the sample. While the cryo-stage (Quorum) can be rotated by 360° and allows tilting as well as xyz motion, thus enabling imaging in a variety of geometries, the best ion extraction can be achieved if the sample plane is perpendicular or near-perpendicular to the ToF axis. During FIB-SIMS imaging, the ToF extraction optics are inserted close to the sample (Extended Data Figure 2b) to allow the application of the extraction voltage near the sample, resulting in an orthogonal deflection of the ions into the ToF analyser. During the imaging process, the SIMS control software (TofDaq, TOFWERK) controls the FIB scan generator to align the FIB scanning with the extraction pulses."

We have also extended the comparison to other techniques by adding a comparison with EELS/EDX and correlative nanoSIMS:

a. To this end, we provide a supplementary table enumerating the molecular ions identified in our SIMS data. As reviewer 3 points out, detecting these molecular ions is not possible in EELS/EDX, which rely on elemental atomic characteristics. This enumeration will help to clearly distinguish our method from such element-based techniques, highlighting the complementary nature of our workflow with previous studies.

Excerpt from Supplementary Table S1:

Ions detected in negative mode, peaks with intensities > 10^{-6} counts/extraction

12	C
13	^{13}C, CH
16	O
17	OH
18	?
19	F
24	C2
25	C_2H
26	CN
27	CHN, ^{13}CN
31	P
32	O_2, S?
33	HO_2?PH_2? HS
34	H_2O_2?
35	Cl
36	C_3
37	Cl isotope
38	C_2N, F_2?
39	C_2HN?
40	CN_2?
41	CHN_2
42	NCO, C_2H_2O, C_2H_4N
43	C_2H_3O, CHNO
45	HCO_2, C_2H_5O
47	PO, CH_3S
48	C_4

49	C_4H
50	C_3N
51	C_3HN?
59	$C_2H_3O_2$
60	C_5
61	HN_2O_2
62	NO_3
63	PO_2

b. To further highlight how cryo-FIB-SIMS is useful, we also provide new experimental data showing the detection of different isotopes of the same element (Extended Data Figure 5c), which is not possible using EELS/EDX.

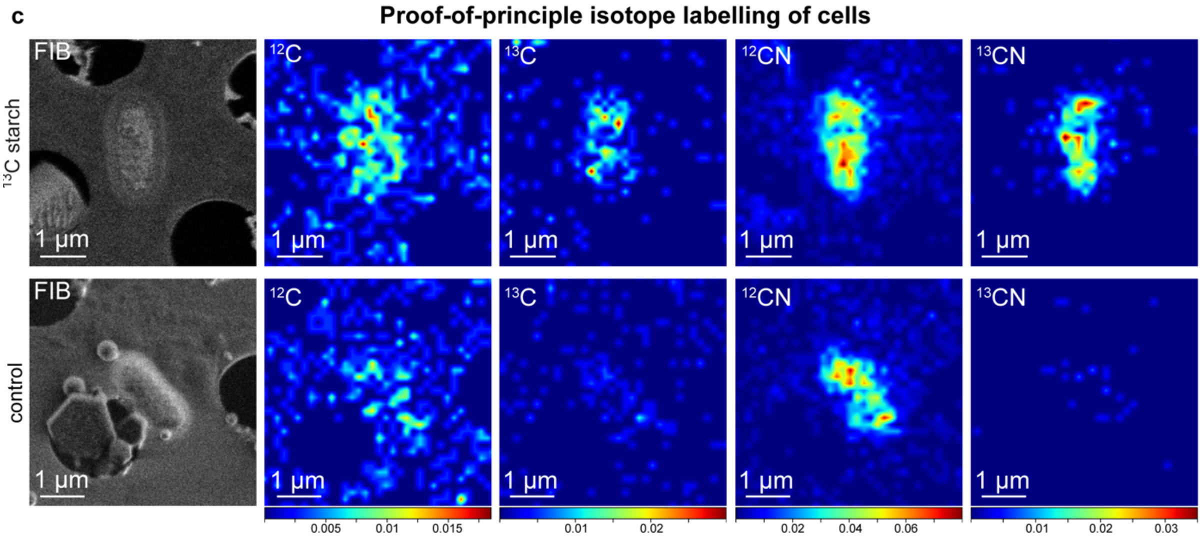

c Cryo-FIB-SIMS imaging of *Bacteroides thetaiotaomicron* in media supplemented with ^{13}C-labelled starch (top) and a control culture grown in unlabelled media with glucose as the carbon source (bottom). On the left, secondary electron FIB images are provided to clarify the spatial localization of the cells. Cells exposed to the ^{13}C-labelled starch show clear intracellular signal of both ^{13}C and ^{13}CN, while the cells in the control culture do not exhibit ^{13}C-related SIMS signals.

Line 107-113:
"As SIMS can distinguish elemental isotopes[12,21,22], our workflow is also compatible with more subtle labels. To illustrate this, we incubated the gut bacterium *Bacteroides thetaiotaomicron* in a medium containing ^{13}C-labelled starch and subsequently imaged the resulting sample by cryo-FIB-SIMS (Extended Data Figure 5c). The data shows strong cellular signals corresponding to both ^{13}C and ^{13}CN, with the presence of ^{13}CN suggesting that the starch had been metabolized to organic molecules containing both C and N. As expected, cells from the control culture did not exhibit these signals."

c. In addition, we provide an in-depth discussion that compares our approach with other chemical imaging techniques, such as EELS/EDX or nanoSIMS, in terms of detectability of elemental and molecular ions including isotopes and spatial imaging capabilities, outlining the relative merits of each technique, which will show how our method is distinctive and allows complementary information to these existing techniques.

Line 242-263:
"These results anchor cryo-EM-FIB-SIMS within the spectrum of spatio-chemical imaging techniques for biological specimens, which ranges from other SIMS-based methods to spectroscopic techniques such as Electron Energy Loss Spectroscopy (EELS) and Energy Dispersive X-ray Spectroscopy (EDX)[15,16,38–43]. While these methods all generate spatially resolved information about chemical composition and molecular localization, they differ vastly in compositional sensitivity and spatial resolution. As SIMS directly detects the secondary ions produced during the interaction between primary ion beam and sample, not only elemental, but also molecular and isotopic secondary

ions can be detected. The detectable mass range depends on the primary ion beam in the first instance, and on the mass resolution and the number of simultaneously detectable ions, which are characteristics of the mass spectrometer, in the second instance. EELS, EDX and Scanning Transmission Electron Microscopy (STEM) detect scattered electrons or photons, hence only data regarding elemental composition, but not about molecules or isotopes, is acquired, making cryo-FIB-SIMS complementary to those methods that offer higher spatial resolution up to the sub-molecular range.
The attainable spatial resolution in SIMS depends on the choice of primary ion beam and the ionisation characteristics (including sputter yield) of the secondary ions being considered. Using monoatomic primary ion beams such as Ga permits high spatial resolution SIMS imaging, allowing the resolution of features up to approximately 50 nm[6,44]. By combining SIMS imaging with low-dose, high-resolution spatial imaging techniques such as cryo-EM and cryo-SEM[16,45] in sequential imaging workflows, the chemical and spatial data can be correlated to enable the nanometer scale localization of elements and molecules within the cellular environment. While cryo-SEM imaging offers compatibility with volumetric imaging on thicker samples, workflows employing cryo-EM or cryo-ET, as presented here, allow correlative imaging at unprecedented spatial resolution at which individual molecules can be resolved."

d. Finally, we have also extended the discussion to highlight future possibilities for the detection of larger molecules. Larger molecules, including whole drug molecules and peptides, could be detected by adapting our workflow to include the use of larger ion beams, which are regularly used in other types of SIMS experiments.

Line 264-273:
"Larger molecules can be tracked in cryo-FIB-SIMS imaging via their elemental (e.g. metals, halogens) or small molecular degradation fragments (Supplementary Table S1) in untagged or labeled specimens[12,21,22,46]. However, the low ionization yield as well as the large interaction depth of the Ga beam with the sample[47] results in a very low probability of extracting peptides, lipids or oligonucleotides. To extend the range of detectable molecules, larger ion beams[9,11,12] such as gas cluster ion beams (GCIB)[4] could be utilized, which would have the added benefit of very precise depth analysis, albeit at the expense of lateral spatial resolution. Further development of the method to encompass correlative cryo-EM and SIMS imaging with a range of different primary ion beams could, in the future, provide comprehensive data on ions of a larger mass range, including peptides, lipids, and drug molecules, while retaining the high spatial resolution characterizing the technique described here."

The authors convincingly demonstrate their workflow on proof of principle samples and in bacteria. However, the authors miss the opportunity to demonstrate a broader applicability for different types of samples. While the authors use different proof-of-principle bacteria samples (bacteria lamella and fluorescently and gold co-labelled samples) the conclusions from those experiments don't necessarily transfer to samples of eukaryotic/mammalian cells or tissue due to the higher complexity (e.g. membrane organelles with different ion concentrations) of such samples. It remains unclear how those expectingly more complex data in both modalities can be interpreted without data demonstrating it.

In order to make this point, I would recommend for the authors to demonstrate their method on an eukaryotic cell sample.

As suggested, we have added proof-of-principle data demonstrating the application of our workflow to lamellae of yeast cells (Fig. 3f, Extended Data Figure 7i), showing differential MS signal from the cytosol and nucleus/vacuoles. We have updated the corresponding text accordingly:

Line 163-166:
"Next, lamellae of eukaryotic cells, namely plunge-frozen *Saccharomyces cerevisiae* yeast cells, were subjected to the cryo-EM-FIB-SIMS workflow (Fig. 3f, Extended Data Figure 7i), demonstrating differential distributions of ions such as K, Na and Mg in different compartments of the cell."

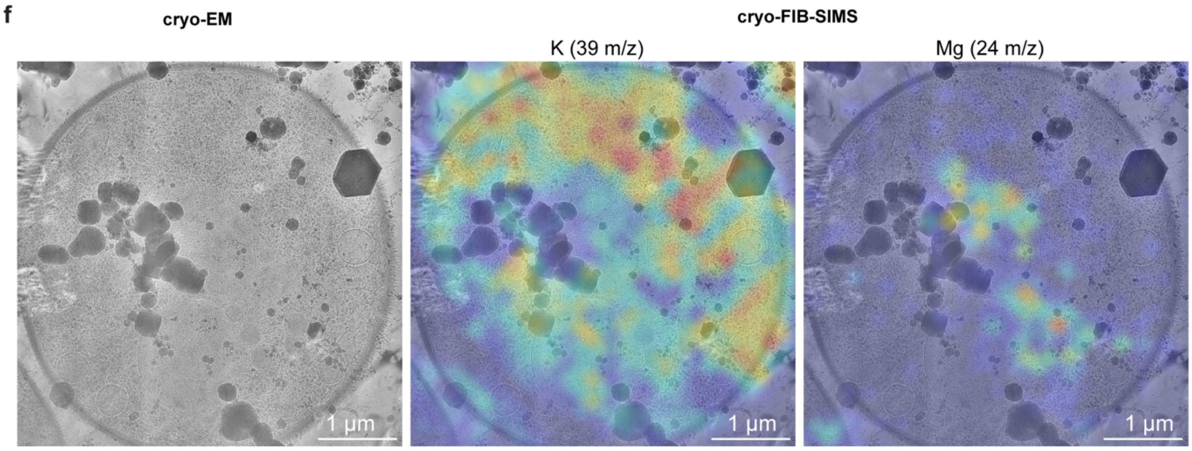

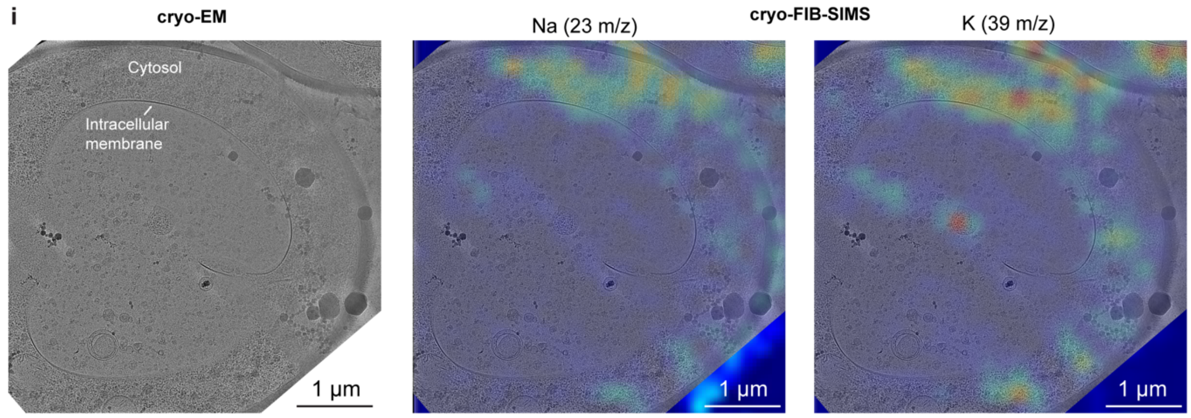

f Cryo-EM-FIB-SIMS workflow applied to cryo-FIB-milled lamellae of yeast cells shows differential signals of Mg and K between cellular compartments, thus illustrating the generalizability of the workflow to eukaryotic samples.

The authors use a proof of principle sample to show that the FIB-SIMS cryo-EM correlation can also be performed when cryoLM is performed for additional correlation. I am curious why the authors decided to demonstrate this on a proof of principle sample instead of a more interesting sample. In my mind, there is no reason why the integration with an additional fluorescent label would provide extra challenges as we know that we can correlate fluorescence with TEM and this study shows that we can correlate FIB-SIMS with TEM and those two modalities are orthogonal. It would have been more interesting if the authors instead had labeled a specific component of their bacterial cells and used fluorescence to guide them there in order to generate new biological data using their correlative FIB-SIMS and TEM approach - ideally in the context of a biological question. Again, this would have been more impactful on eukaryotic samples - where due to the higher complexity - the guidance from the fluorescence is more likely needed.

Since this is a first demonstration of the technique, we wanted to work with a known and previously characterised specimen allowing for clear labelling for both cryo-LM and cryo-FIB-SIMS. We agree that this could be extended in the future to other more biologically focused questions. We have extended the discussion to highlight this:

Line 230-233:
"Using the full cryo-CLEM-FIB-SIMS workflow will be especially beneficial when studying thicker and more complex samples, such as eukaryotic cells or multicellular tissue-like specimens, as fluorescence can be used to guide lamellae milling as well as site-specific cryo-EM and cryo-FIB-SIMS imaging."

We also agree that many people care deeply about eukaryotic specimens, which is why we have updated the manuscript with data from yeast cells (see above).

Finally, the authors study bioaccumulation of BPAF into an environmental bacterium C. crescentus. The authors clearly demonstrate the effect on growth and proteomic changes, including the upregulation of bacterial efflux pump related proteins and demonstrate that BPAF is getting taken up into storage granules. This part of the

manuscript could get further improved by making the motivation of the specifics of this study more clear eg by providing more details in the introduction and discussion on how studying bioaccumulation of BPAF in this bacterium could potentially help with the challenges this pollutant poses to humans and the environment and what the presented data mean in this context.

We have included additional details to this part of the manuscript as requested. Specifically, we think that understanding the effect on organisms exposed to BPAF in the environment is an important first step, setting the stage for future studies that explore the effect of BPAF and related compounds on other organisms such as gut bacteria and mammalian cells.

Line 172-175:
"Gaining an understanding of the effect of BPAF-exposure on organisms in the environment is an important first step towards future explorations of the impact of BPAF and related compounds on the human body as well as wider ecosystems, from both the host and host-associated microbiome perspective."

The manuscript is overall well written and the data are clearly presented. The authors indicate sample sizes and statistical tests in their method section and included convincing cell numbers for all experiments.

Thank you for your consideration, we really appreciate it.

Minor comments:

The number of prepared samples is mentioned throughout the method sections. The authors could consider adding that information to the section on cell numbers from all experiments (lines 580-587) to specifically indicate the number of different samples those cell numbers are from.

We have added this to the relevant section to make the information more readily available.

Line 709-718:
"The following numbers of cells were analyzed in the cryo-FIB-SIMS experiments shown in this study: Gold nanoparticle labelled *C. crescentus* (Fig. 1b) n=24 on two separately prepared grids; unlabelled *C. crescentus* (Fig. 2): n=105 on three grids from separately prepared cultures; cryo-CLEM-FIB-SIMS: mRFP+Au-labelled (Fig. 3): n=24, GFP-labelled: n=29 from two separate labelling experiments; BPAF uptake (Fig. 5): n=162 BPAF-exposed, n=50 unexposed imaged on a total of 8 grids from three biological replicates. Storage granule size measurements have been carried out in Fiji [59] from cryo-EM images for n=1,464 granules in BPAF-exposed cells, n=252 in unexposed cells, and statistical tests (unpaired t-test) have been performed using GraphPad Prism version 10.4.1. For the cryo-FIB-SIMS experiments on lamellae, 15 lamellae of concentrated *P. aeruginosa* and 20 lamellae of yeast cells were imaged from two independent biological replicates."

The authors discuss the 3D capabilities of the FIB SIMS approach and show 3D SIMS and a tomogram in their extended data videos but don't show any 3D overlays. Please provide a 3D overlay of the 3D FIB SIM data onto a tomogram or comment if this presents additional challenges.

We have now provided an overlay of the tomographic slice in Fig 3e with the corresponding FIB-SIMS data to partly address the reviewer's concern. A a full 3D overlay is currently challenging due to inaccuracies in measuring the exact "Z"-thickness of FIB-SIMS slices, differences in robustness and sputtering rates of features (e.g. some of the granules are milled away slower than the surrounding cellular environment), which can result in distortions of the "Z"-distributions, and, depending on sample type, on the movement of the support film under ion beam irradiation. The tomograms in the extended data videos serve the purpose of giving a clearer impression of the different types of aggregates observed in the BPAF-exposed cells, we have not collected cryo-FIB-SIMS data on these specific cells.

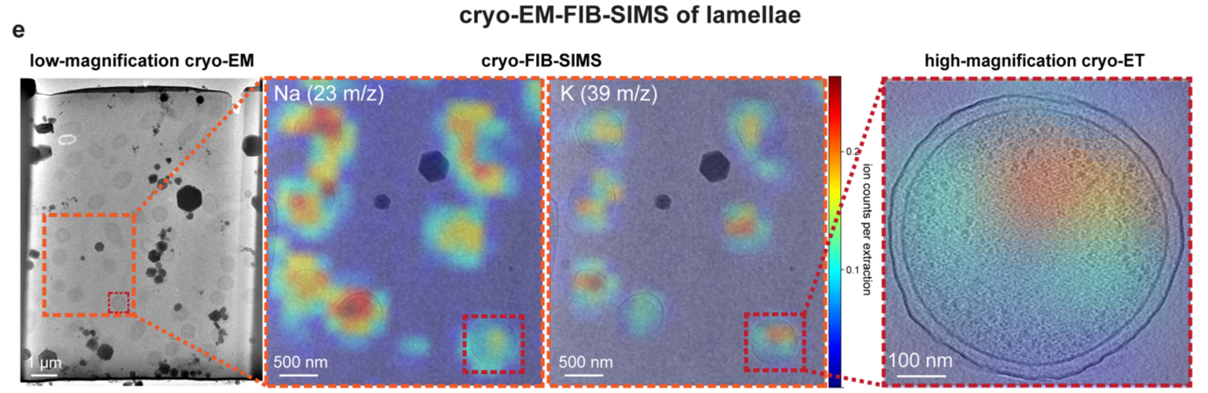

e Cryo-EM-FIB-SIMS workflow applied to cryo-FIB-milled lamellae containing *P. aeruginosa* bacteria and further correlated with cryo-ET of FIB-milled cells. The high-magnification cryo-ET image is a slice from a tomogram.

Please provide more details on how the image to image (TEM to FIB-SIMS) correlation is done. The authors mention that they include FIB images for the correlation in the caption of Figure 1 but don't provide more details. Is it just done manually and visually based on the outline of the cell shape visible in the FIB image? How precise is the correlation? How would you do it in 3D?

We have added a more detailed description of this process to the methods section as requested. In brief, we manually re-scale the cryo-EM data and the FIB-SIMS data (which includes both the spatial FIB image from secondary electron detection and the SIMS data) to the same scale and use larger spatial features discernible in both types of data to perform the overlay. These features are outlines of bacterial cells and the holes in the cryo-EM grid.

Line 702-708:
"To overlay cryo-EM and cryo-FIB-SIMS data, we scale the cryo-EM data and the FIB-SIMS data (which includes both the spatial FIB image from secondary electron detection and the SIMS data) to the same size. As spatial features are generally more clearly discernible in the secondary electron FIB image, we manually overlay the cryo-EM and the FIB images, using larger features such as the cellular outline and larger intracellular aggregates as well as the holes in the carbon support film. As the FIB and SIMS images are collected simultaneously and with the same field of view and pixel size, the subsequent overlay with the SIMS data is straightforward."

Figure 1: From the schematic in Fig 1a, the workflow is not immediately clear. The schematics as presented in Extended data figure 1 a-c seem more intuitive. The authors could consider generating a workflow figure that combines elements from figure 1 and extended data figure 1 to present their workflow in a more intuitive way in a main figure.

We think that adding a new Extended Data Figure 2 that includes pictures of our setup, along with the associated text will make our workflow clear to readers. We prefer to keep the schematic in the main figure and the cartoon in the supplement. We hope this is OK.

Figure 2, caption: the authors mention that gold is added to the sample - the reviewer is assuming this is added to help with alignment of potential tilt series - or is there another purpose? Please clarify and comment.

The reviewer's assumption is correct; the gold was added to provide fiducials for potential tomographic imaging and reconstruction. We have clarified this in the figure caption.

Line 457-458:
"The black dots are 10 nm protein-A-gold fiducials added to this otherwise untagged specimen for alignment of tilt series (cryo-ET data)."

In line 63: "Multicellular, tissue like specimens" and line 208,209: "multicellular specimens" is misleading - given that the authors perform work on bacteria. I believe the authors refer to their showcase of a lamella from multiple bacteria in solution. The authors should specify that their lamella is on multiple bacteria or include data on mammalian cells or tissue as discussed above.

We have clarified this in the manuscript and in addition provide data collected on eukaryotic yeast cells as discussed above.

Line 59-62:
"We provide proof-of-principle data for tagged and untagged biological specimens and extend our approach to show that the technique is compatible with cryo-light microscopy (cryo-LM), as well as with thicker specimens, such as eukaryotic cells, which can be imaged in the form of FIB-milled lamellae."

Line 115-188: The authors observe different ion distribution in the bacterium but don't interpret or comment further on those observations. The authors could comment if this observed differential distribution, perhaps in particular the increased Mg concentration in storage granules, has been expected based on previous findings or if those are new or unexpected findings.

While the general literature on polyphosphate granules suggests that calcium, potassium and magnesium could serve as counterions to polyphosphate chains and thus be present within the granules (e.g. Bonting et al., Arch Microbiol (1993) 159:428-434, Röske et al. Int. Revue ges. Hydrobiol. Hydrogr. (1995) 80: 605-621, Tocheva et al. J Bacteriol. 2013;195(17):3940-6), we have not found specific literature regarding the full ionic content of storage granules in *Caulobacter crescentus*. We have commented on this in the main text and cited the relevant studies.

Line 127-129:
"This is in line with previous studies on storage granules in different bacteria, which suggest that potassium, magnesium and calcium could act as counterions to polyphosphate chains within storage granules[23–25]."

Line 120: From the characterization of bacteria cells by cryo-SIMS the authors conclude a correlation between TEM contrast and SIMS peak strength. How confident are the authors about this? I would recommend that the authors provide other examples from their data to strengthen that conclusion, provide more reasoning for that statement or otherwise remove that statement.

We have removed this statement, because further work is needed to establish the correlation. We agree that this needs careful experimentation on some control specimens.

Line 182 and figure 5: the authors comment on the presence of "high-contrast, often irregularly shaped features" that are not "commonly observed in C. crescentus" but don't provide any further observations or interpretations of those. The authors should comment if they observed any specific signal in their cryo-FIB-SIMS data for those.

We have not observed specific SIMS signal for the other type of aggregates, we have commented on this in the manuscript.

Line 206-208:
"The lack of specific SIMS signal from the high-contrast granules suggests that their formation might be part of a generalized stress response from the cell."

The authors could consider providing an image or schematic of the technical details of the instrument to make the instrumental design more clear.

We have provided a photographic image of the inside and outside the instrument as a new Extended data figure 2 (see above).

Extended figure 1: Please label elements in the spectrum - even though it is already color coded based on annotations in images below - an additional label would make it more obvious.

We have added the labels to the spectrum as requested.

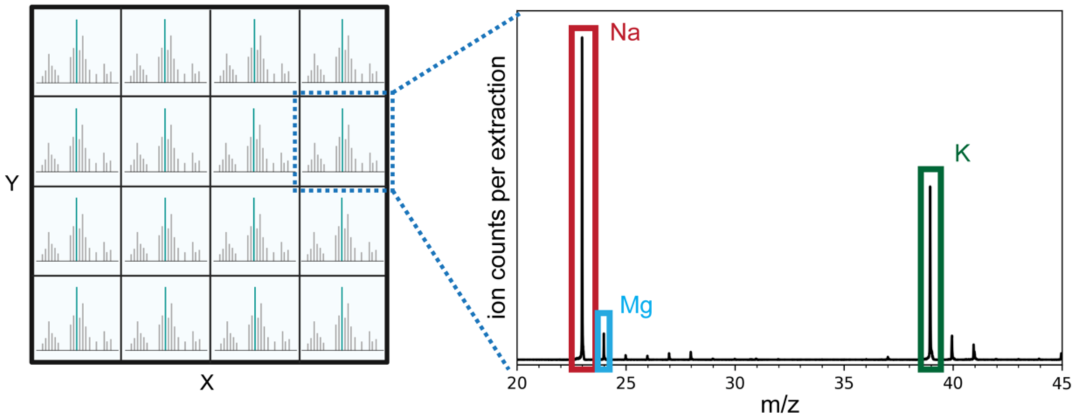

Extended figure 1 caption d) Please explain what is shown.

We have added an extended caption stating that Figure S1d shows an example of SIMS data for selected elemental peaks collected on a *C. crescentus* cell.

"**d** Positive ion mode correlative analysis of a *C. crescentus* cell showing the cryo-FIB-SIMS data of three prominent *m/z* peaks (Na, Mg, and K, top) as well as overlays with the cryo-EM image (bottom). The colour scale of the MS images corresponds to the detected ion count per extraction."

Referee #2 (Remarks to the Author):

Ochner et al. present a manuscript on the sub-cellular chemical mapping in bacteria, using correlated cryo-EM and Mass Spec.

This was achieved by freezing bacterial samples on cryo-EM grids (plunge-freezing or HP-freezing), followed by first cryo-EM or cryo-ET imaging, and then cryo-FIB-SIMS imaging. For the latter, the 30kV Ga beam in the FIB-SEM is used to blast away surface ions from the frozen sample, which are accelerated with either a positive or negative voltage, and the correctly charged ions are them quantified with a TOF mass spec directly inside the SEM.

The combined application of cryo-EM (or cryo-CLEM) with FIB-SIMS is unique and will certainly be very useful for many biomedical applications. Bacteria are tiny compared to other cells. If it is possible to localize different ions, such as F, PO2, Mg or BPAF, directly within aggregates inside of bacteria, then it should also be possible to extend this to lamellae of human tissue, e.g., brain in neurodegeneration or cancer tissue, to localize specific signatures of elements, such as prionoid fibrils and what is associated with them (Fe?), or nanoparticles or other small labels. The method should have a very high value for medical research.

Thank you for your encouraging comments, we appreciate the care taken by the reviewer in assessing our work.

Details in the implementation remain vague, though.

How is the geometry of the FIB-SIMS imaging implemented? Does the Ga beam scan over the sample from the vertical top to be able to record an image?

This was also a concern from reviewer 1; we have added a photograph of the inside of the microscope chamber as a new Extended Data Figure 2 to clarify the instrument geometry and have extended the methods section to describe this in more detail. While the stage setup is flexible and allows both 360° rotation as well as tilting (in addition to extensive xyz-motion), during SIMS imaging, both the ion beam and the mass spectrometer are at an angle close to 90° to the sample surface (although imaging at other angles is possible if a specific application/sample would require that).

Line 675-679:
"The imaging geometry is of the cryo-FIB-SIMS instrument is depicted in Extended Data Figure 2. The FIB column and the ToF mass spectrometer have nearly the same angular orientation with respect to the sample. While the cryo-stage (Quorum) can be rotated by 360° and allows tilting as well as xyz motion, thus enabling imaging in a variety of geometries, the best ion extraction can be achieved if the sample plane is perpendicular or near-perpendicular to the ToF axis."

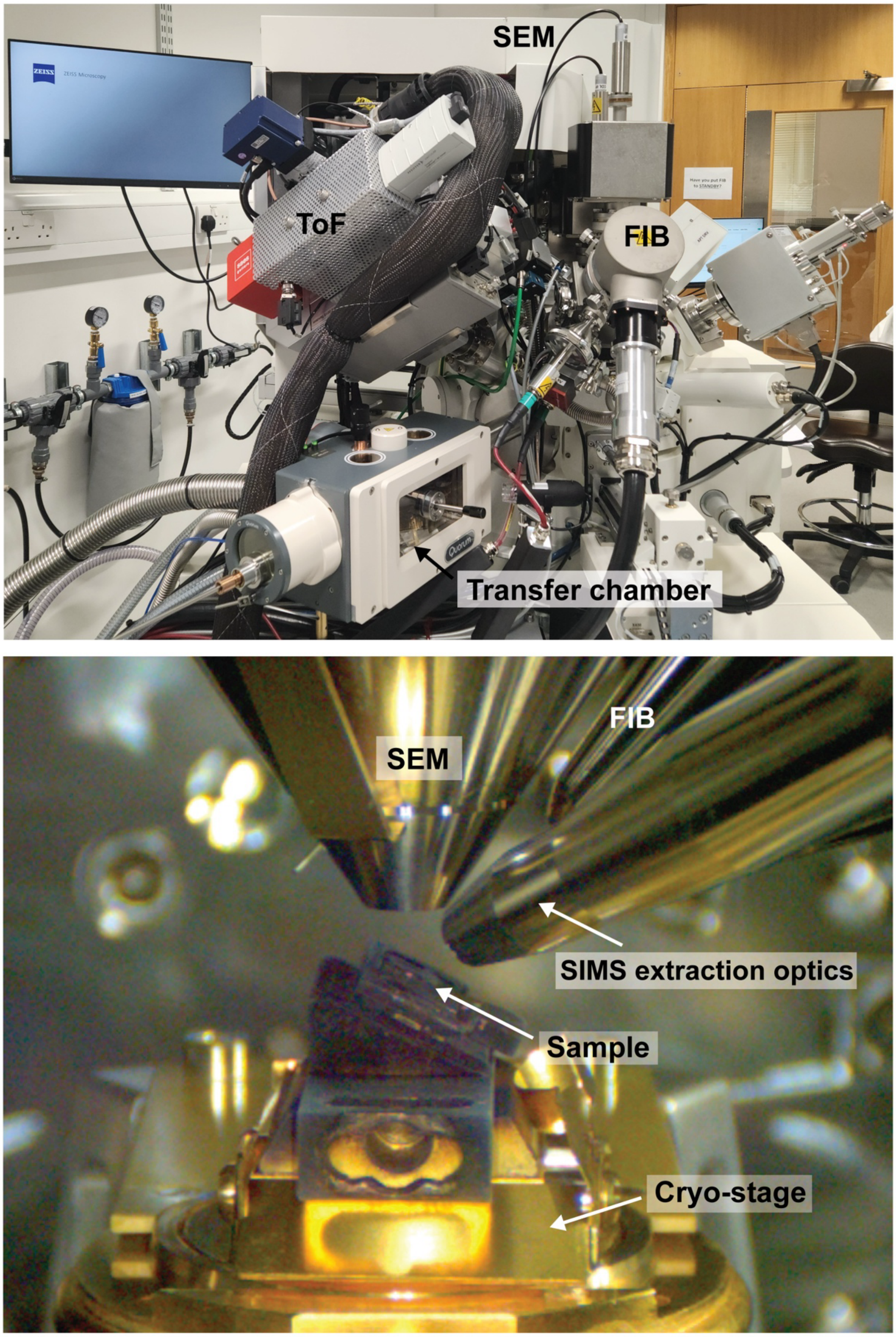

Extended Data Fig. 2: Cryo-FIB-SIMS instrument setup. a Photograph of the outside of the machine with relevant components marked. **b** Photograph of the inside of the microscope chamber with SIMS extraction optics inserted. Relevant components have been highlighted.

If so, how is the penetration depth of the beam regulated?

The penetration depth depends on various factors and can be regulated via the beam parameters (current/voltage/dwell time). We have added these details to the methods section.

Line 690-693:
"The penetration depth depends on various factors such as beam current, voltage and dwell time. If the sample were homogeneous, homogeneous ablation should be achieved, however, due to different features within the sample interacting differently with the primary ion beam as well as beam damage artifacts, inhomogeneities during ablation can occur."

How do the authors achieve a homogeneous ablation of the surface, so that a 3D stack is recorded? Or don't they achieve this?

Sorry if this was not clear, we have clarified this in the methods section now. Briefly, a 3D stack is recorded by sequential scanning over the same area (with the same parameters). While this should result in homogeneous ablation if the sample can be assumed to be homogeneous, there are some restrictions as different features within the sample might interact differently with the ion beam, leading to potential inhomogeneities during ablation.

Line 690-693:
"The penetration depth depends on various factors such as beam current, voltage and dwell time. If the sample were homogeneous, homogeneous ablation should be achieved, however, due to different features within the sample interacting differently with the primary ion beam as well as beam damage artifacts, inhomogeneities during ablation can occur."

Where is the voltage applied between the sample and the MS? How is the MS coupled to the SEM? Extended Data Fig. 1b is merely a scheme. A photo or a better scheme would be helpful.

We have clarified this in the methods section now, as requested. The SIMS ion optics can be inserted close to the sample, the extraction voltage pulses are applied at the front of the ion optics column, resulting in an orthogonal deflection of the ions into the ToF analyser. In terms of the actual imaging process, the SIMS control software is used to control the FIB scan generator to align the FIB scanning with the extraction pulses.

Line 679-683:
"During FIB-SIMS imaging, the ToF extraction optics are inserted close to the sample (Extended Data Figure 2b) to allow the application of the extraction voltage near the sample, resulting in an orthogonal deflection of the ions into the ToF analyser. During the imaging process, the SIMS control software (TofDaq, TOFWERK) controls the FIB scan generator to align the FIB scanning with the extraction pulses."

How is correlation between the signals of cryo-EM and SIMS achieved?

We have provided more details in the revised manuscript as requested. Please also see our response to reviewer 1's comment above.

Line 702-708:
"To overlay cryo-EM and cryo-FIB-SIMS data, we scale the cryo-EM data and the FIB-SIMS data (which includes both the spatial FIB image from secondary electron detection and the SIMS data) to the same size. As spatial features are generally more clearly discernible in the secondary electron FIB image, we manually overlay the cryo-EM and the FIB images, using larger features such as the cellular outline and larger intracellular aggregates as well as the holes in the carbon support film. As the FIB and SIMS images are collected simultaneously and with the same field of view and pixel size, the subsequent overlay with the SIMS data is straightforward."

The cryo-EM image in Fig. 1b left suggests from the inset that there are many gold particles. If that inset is a cross-section, then that would suggest that the bacterial surface is densely covered with gold. However, the FIB-SIMS image in Fig. 1b right shows only a hand-full of gold particles that are heterogeneously scattered over the surface. How can these two images be aligned? How do the authors explain the density difference?

Regarding the specific question concerning Figure 1b, the inset is a high-magnification two-dimensional cryo-EM projection image of the region marked by the red box. According to the cryo-EM data, the bacterial cell surface should indeed be covered in gold. However, when correlating to the SIMS data, one must bear in mind the difference in spatial resolution and sampling between those data sets which does not allow the us to resolve

individual gold nanoparticles (diameter 5 nm) in the SIMS data. Hence, a dot in the Au-channel of the SIMS data corresponds to a larger area of the cell surface and will include contributions of several gold nanoparticles and thus no direct comparison of the density of features in the two imaging modalities is possible. The actual correlation has been done via larger features such as the cell shape. We realise that this could be confusing for readers, therefore we have added some text in the manuscript to clarify.

Line 442-444:
"Discrepancies between the cryo-FIB-SIMS gold signal and the distribution of gold nanoparticles in the cryo-EM image are likely the result of the difference in resolution between the two imaging modalities as well as nonuniform ionization yields across the cell."

Which particles are detected in the FIB-SIMS? If Na, K, Mg and Au are detected atomically, and PO2 and PO3 as ions, which other elements are detectable as molecules or atoms? An outlook for which medically relevant complexes could be detected, would be a valuable addition.

We have extended our manuscript regarding this important question. To highlight what we have already detected, we provide a new Supplementary table S1 showing what secondary ion peaks we could observe.

Excerpt from Supplementary Table S1:

Ions detected in negative mode, peaks with intensities > 10^{-6} counts/extraction

12	C
13	^{13}C, CH
16	O
17	OH
18	?
19	F
24	C2
25	C_2H
26	CN
27	CHN, ^{13}CN
31	P
32	O_2, S?
33	HO_2? HS?
34	H_2O_2?
35	Cl
36	C_3
37	Cl isotope
38	C_2N, F_2?
39	C_2HN?
40	CN_2?
41	CHN_2
42	NCO, C_2H_2O, C_2H_4N
43	C_2H_3O, CHNO
45	HCO_2, C_2H_5O
47	PO, CH_3S
48	C_4
49	C_4H

50	C_3N
51	C_3HN?
59	$C_2H_3O_2$
60	C_5
61	HN_2O_2
62	NO_3
63	PO_2

In short, the ionisation probability of molecular fragments strongly depends on the interaction between the sample and the Ga beam, which favours the generation of smaller ions. However, many medically relevant compounds can be detected via specific elements they contain (e.g. halogens, metals) or via specific fragments detectable on our FIB-SIMS system. Finally, the use of larger ion beams allows for the detection of larger fragments, albeit at the expense of spatial resolution. One important focus for future method development will therefore be on the use of larger ion beams. We have extended the discussion to make these points clear.

Line 264-273:
"Larger molecules can be tracked in cryo-FIB-SIMS imaging via their elemental (e.g. metals, halogens) or small molecular degradation fragments (Supplementary Table S1) in untagged or labeled specimens[12,21,22,46]. However, the low ionization yield as well as the large interaction depth of the Ga beam with the sample[47] results in a very low probability of extracting peptides, lipids or oligonucleotides. To extend the range of detectable molecules, larger ion beams[9,11,12] such as gas cluster ion beams (GCIB)[4] could be utilized, which would have the added benefit of very precise depth analysis, albeit at the expense of lateral spatial resolution. Further development of the method to encompass correlative cryo-EM and SIMS imaging with a range of different primary ion beams could, in the future, provide comprehensive data on ions of a larger mass range, including peptides, lipids, and drug molecules, while retaining the high spatial resolution characterizing the technique described here."

In the figures, it is not clear, which panels show the recorded images (e.g., the inset in Fig. 1b left), and which images are slices from within reconstructed volumes (e.g., Fig. 3e right is certainly a slice).

All the cryo-EM images shown, with the exception of Fig. 3e right, are 2D projection images. The reviewer is absolutely correct that Fig. 3e right is a slice from a tomogram. We have updated text and figure legends to clearly state this.

Line 474-475:
"The high-magnification cryo-ET image is a slice from a tomogram."

The fonts in Fig. 4a are too small.

We have increased the font size in Fig. 4a.

PYE should also be defined in the Method section, not only in the figure legends.

We have updated the relevant parts of the methods section accordingly.

Referee #3 (Remarks to the Author):

In their well-written manuscript titled

"Sub-cellular chemical mapping in bacteria using correlated cryogenic electron and mass spectrometry imaging"

submitted for publication in Nature Dr Bharat and co-authors demonstrate a novel workflow to investigate structure and chemical composition of bacteria at a sub-cellular level.
Correlative structural analysis (by electron microscopy) and chemical mapping (using ToF-SIMS) of microbes under cryogenic conditions is certainly a very interesting approach for the community of micro-biologists working in different fields, e.g. microbial ecology, bio-technology and environmental micro-biology to name a few.
Of course, several approaches to combine structural and chemical micro-analytics on biological systems have already been published, however, these are typically conducted at room-temperature which requires chemical preparation of the samples prior to imaging and thus modifies the chemical composition of cells or tissues.
The next logic step - which the authors successfully made - is to develop such a workflow under cryogenic conditions using vitrified water-ice as embedding medium thus avoiding the need for chemical fixation.
However, they are not the first to do so.
The number of published reports on correlative cryo-imaging and chemical mapping is small but there are for instance the reports by
- De Castro et al. in Microscopy and Microanalysis, 29 (2023), DOI: 10.1093/micmic/ozad067.390 who employed cryo-imaging and SIMS in a scanning helium ion microscope on a plunge-frozen cell culture or by
- Meibom et al. in BMC Biology (2023), DOI: 10.1186/s12915-023-01623-0 who used cryo-SEM and cryo-NanoSIMS to image biological tissues.
While the authors may not have been aware of the publication by Meibom et al. they correctly cite De Castro et al. in their manuscript.
In my opinion, a discussion of the pros and cons of their workflow in comparison with the approaches published by these groups is missing an should be added to the manuscript.

We thank the reviewer for these comments. We certainly did not want to disrespect these eminent past studies, which is why we had cited several of the above papers already. To ensure that there is no confusion, we have also cited the other papers mentioned.

15. De Castro, O. *et al.* npSCOPE: A New Multimodal Instrument for In Situ Correlative Analysis of Nanoparticles. *Anal. Chem.* **93**, 14417–14424 (2021).
16. Meibom, A. *et al.* Correlated cryo-SEM and CryoNanoSIMS imaging of biological tissue. *BMC Biol* **21**, 126 (2023).

We have also extensively discussed our study with previous efforts. We believe that our work is highly complementary to studies from experts in the chemical imaging field.

Line 258-263:
"By combining SIMS imaging with low-dose, high-resolution spatial imaging techniques such as cryo-EM and cryo-SEM[16,45] in sequential imaging workflows, the chemical and spatial data can be correlated to enable the nanometer scale localization of elements and molecules within the cellular environment. While cryo-SEM imaging offers compatibility with volumetric imaging on thicker samples, workflows employing cryo-EM or cryo-ET, as presented here, allow correlative imaging at unprecedented spatial resolution at which individual molecules can be resolved."

The novelty in the work of Dr Bharat et al. could be that a time-of-flight SIMS instead of a sector-field spectrometer has been used and that the method also allows for cryo-light microscopy.
In that light, the presented workflow has the potential to become another powerful standard tool in the belt of techniques for micro-analysis of microbes at sub-cellular level.
Without any doubt, this versatile correlative cryo-SEM-SIMS workflow opens up doors for understanding the chemistry of cells at sub-cellular level.

Thank you for highlighting these novel aspects of our methodology. While there are some similarities to previous contributions on the SIMS side (although, as correctly pointed out by the reviewer, we are using a different type of mass spectrometer), our workflow, as far as we are aware, is the first one to fully incorporate cryo-EM/ET imaging (which allows higher spatial resolution imaging than SEM) and has the capability to incorporate other techniques established in the field, such as cryo-CLEM and FIB-milling of lamellae. We have used the reviewer's comments to modify the discussion.

Line 258-263:

“By combining SIMS imaging with low-dose, high-resolution spatial imaging techniques such as cryo-EM and cryo-SEM[16,45] in sequential imaging workflows, the chemical and spatial data can be correlated to enable the nanometer scale localization of elements and molecules within the cellular environment. While cryo-SEM imaging offers compatibility with volumetric imaging on thicker samples, workflows employing cryo-EM or cryo-ET, as presented here, allow correlative imaging at unprecedented spatial resolution at which individual molecules can be resolved.”

Line 227-233:
“The versatility of the workflow, achieved by the combination of various imaging modalities, opens up a wide range of future applications, such as the sub-cellular (sub-organellar in eukaryotes) localization of molecules of interest or the targeted exploration of local morphology and chemical composition of subcellular features in both prokaryotic and eukaryotic cells. Using the full cryo-CLEM-FIB-SIMS workflow will be especially beneficial when studying thicker and more complex samples, such as eukaryotic cells or multicellular tissue-like specimens, as fluorescence can be used to guide lamellae milling as well as site-specific cryo-EM and cryo-FIB-SIMS imaging.”

However, based on the presented data it appears that so far the major application (so far) is to measure and visualize distributions of chemical elements in the cells.
For that, of course, also an energy-dispersive X-ray spectrometer (EDS) could be coupled to the SEM instead (or even in addition) to the ToF-SIMS spectrometer.
It would be nice if the authors could mention this possibility in the manuscript and together with a short comparison of the two methods.

As suggested, we have discussed how our study differs from EDS and also why overlaying cryo-TEM, rather than SEM, is advantageous to boost spatial resolution.

Line 242-263:
“These results anchor cryo-EM-FIB-SIMS within the spectrum of spatio-chemical imaging techniques for biological specimens, which ranges from other SIMS-based methods to spectroscopic techniques such as Electron Energy Loss Spectroscopy (EELS) and Energy Dispersive X-ray Spectroscopy (EDX)[15,16,38–43]. While these methods all generate spatially resolved information about chemical composition and molecular localization, they differ vastly in compositional sensitivity and spatial resolution. As SIMS directly detects the secondary ions produced during the interaction between primary ion beam and sample, not only elemental, but also molecular and isotopic secondary ions can be detected. The detectable mass range depends on the primary ion beam in the first instance, and on the mass resolution and the number of simultaneously detectable ions, which are characteristics of the mass spectrometer, in the second instance. EELS, EDX and Scanning Transmission Electron Microscopy (STEM) detect scattered electrons or photons, hence only data regarding elemental composition, but not about molecules or isotopes, is acquired, making cryo-FIB-SIMS complementary to those methods that offer higher spatial resolution up to the sub-molecular range.
The attainable spatial resolution in SIMS depends on the choice of primary ion beam and the ionisation characteristics (including sputter yield) of the secondary ions being considered. Using monoatomic primary ion beams such as Ga permits high spatial resolution SIMS imaging, allowing the resolution of features up to approximately 50 nm[6,44]. By combining SIMS imaging with low-dose, high-resolution spatial imaging techniques such as cryo-EM and cryo-SEM[16,45] in sequential imaging workflows, the chemical and spatial data can be correlated to enable the nanometer scale localization of elements and molecules within the cellular environment. While cryo-SEM imaging offers compatibility with volumetric imaging on thicker samples, workflows employing cryo-EM or cryo-ET, as presented here, allow correlative imaging at unprecedented spatial resolution at which individual molecules can be resolved.”

For some of the applications shown here, an X-ray spectrometer instead of a ToF-SIMS instrument could indeed yield similar results, however, the ToF-SIMS can detect larger molecular fragments as well as isotopes. We have highlighted this in new Supplementary Table S1 (see below). As outlined in the response to reviewer #1 above, to distinguish our technique from EDS/EELS-based techniques, we provide a list of molecular ions observed in our SIMS data.

Excerpt from Supplementary Table S1:

Ions detected in negative mode, peaks with intensities > 10^{-6} counts/extraction

12	C
13	^{13}C, CH
16	O

17	OH
18	?
19	F
24	C2
25	C_2H
26	CN
27	CHN, ^{13}CN
31	P
32	O_2, S?
33	HO_2? HS?
34	H_2O_2?
35	Cl
36	C_3
37	Cl isotope
38	C_2N, F_2?
39	C_2HN?
40	CN_2?
41	CHN_2
42	NCO, C_2H_2O, C_2H_4N
43	C_2H_3O, CHNO
45	HCO_2, C_2H_5O
47	PO, CH_3S
48	C_4
49	C_4H
50	C_3N
51	C_3HN?
59	$C_2H_3O_2$
60	C_5
61	HN_2O_2
62	NO_3
63	PO_2

In contrast to EDS, naturally, ToF-SIMS has the advantage to be sensitive to isotopes which in turn allows for investigating matter-fluxes by employing isotope-labelling.
Although the authors have not included such data in their manuscript this possibility should be mentioned as it is an important information for the community.

Thank you for this important suggestion. We have added new data showing isotope labelling in the new Extended Data Figure 5c and included a more detailed discussion on isotope labelling to the relevant parts of the main text as mentioned above (see response to reviewer #1).

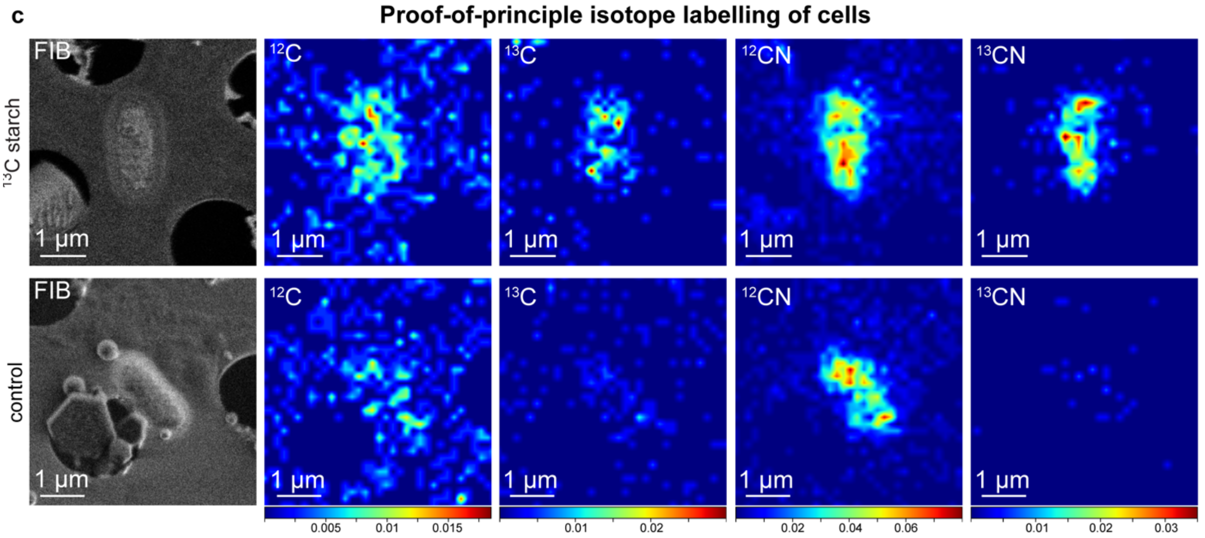

c Cryo-FIB-SIMS imaging of *Bacteroides thetaioataomicron* in media supplemented with ^{13}C-labelled starch (top) and a control culture grown in unlabelled media with glucose as the carbon source (bottom). On the left, secondary electron FIB images are provided to clarify the spatial localization of the cells. Cells exposed to the ^{13}C-labelled starch show clear intracellular signal of both ^{13}C and ^{13}CN, while the cells in the control culture do not exhibit ^{13}C-related SIMS signals.

Line 107-113:

"As SIMS can distinguish elemental isotopes[12,21,22], our workflow is also compatible with more subtle labels. To illustrate this, we incubated the gut bacterium *Bacteroides thetaioataomicron* in a medium containing ^{13}C-labelled starch and subsequently imaged the resulting sample by cryo-FIB-SIMS (Extended Data Figure 5c). The data shows strong cellular signals corresponding to both ^{13}C and ^{13}CN, with the presence of ^{13}CN suggesting that the starch had been metabolized to organic molecules containing both C and N. As expected, cells from the control culture did not exhibit these signals."

I also wonder if in addition to tracing mass 19 (fluorine ions) in the fluorine-rich granules in the Bisphenol-AF-treated cells it would be possible to detect molecular fragments of Bisphenol-AF.
If that were possible, the range of future applications would be tremendously broadened since in that case similar studies with other pollutants without intrinsic element labels could be conducted using this work-flow.
I believe that the manuscript would greatly benefit if the authors could comment on that possibility.
Of course it would be even better and more convincing, if the authors could extract some data of molecular fragments from the existing ToF-SIMS spectra that are the basis for Fig. 5b and amend Fig 5 with such a map.

We have clarified what could be detected in our mass spectra in the text. In principle, detecting molecular fragments of BPAF is possible, even on our current setup, however, this is limited by the low ionisation probability of these fragments during the interaction with the Ga beam. While we do observe some small peaks in the recorded mass spectra that could correspond to BPAF fragments (e.g. CF_3, 69 *m/z,* highlighted in pink in Supplementary Table S1), the signal is not strong enough to allow for a clear spatial visualisation. Hence, fragment detectability currently strongly depends on the compound of interest, its fragmentation pathways when interacting with the Ga beam and the ionisation yield of the respective fragments. However, future instrumentation development will likely include the addition of larger primary ion beams, which will allow for the detection of larger ions and thus molecular fragments.

Line 264-273:

"Larger molecules can be tracked in cryo-FIB-SIMS imaging via their elemental (e.g. metals, halogens) or small molecular degradation fragments (Supplementary Table S1) in untagged or labeled specimens[12,21,22,46]. However, the low ionization yield as well as the large interaction depth of the Ga beam with the sample[47] results in a very low probability of extracting peptides, lipids or oligonucleotides. To extend the range of detectable molecules, larger ion beams[9,11,12] such as gas cluster ion beams (GCIB)[4] could be utilized, which would have the added benefit of very precise depth analysis, albeit at the expense of lateral spatial resolution. Further development of the method to encompass correlative cryo-EM and SIMS imaging with a range of different primary ion beams could, in the future, provide comprehensive data on ions of a larger mass range, including peptides, lipids, and drug molecules, while retaining the high spatial resolution characterizing the technique described here."

Overall, I believe that if the authors could better highlight the novelty of their work and be more convincing with

respect to the unique selling point(s) of this approach the manuscript may be suitable for publication in Nature. I can see the vast potential of the workflow; for instance it could allow for isotope-labelling experiments or it might be possible to detect chemical compounds in microbial cells beyond a mere elemental analysis (here fluorine served as marker) but rather making use of mass-spectra that can be obtained from the ToF-spectrometer. However, to be convincing the authors will need to furnish proof that this is really possible.

Thank you for helping us improve our presentation. We agree, and we have highlighted this in the revised manuscript. We agree that there is a lot of potential to be explored, both using the current setup, which allows for the detection of compounds that include halogens or metals, as demonstrated here, but also for isotope labelling and other labelling strategies. In addition to explorations on the sample and instrumentation side, we also envision future work to lead to progress in analysis software, including advances in extracting data from weaker signals within the mass spectra. We have discussed these possibilities in the new Discussion section, please see above.

Point-by-point response

Reviewer #1 (Remarks to the Author):

In the resubmitted manuscript by Ochner et al., the authors have added substantial new data and strengthened the discussion and description of their workflow, which significantly improves their work. They have thoughtfully addressed the major and minor points raised by the reviewers. In particular, the manuscript now much more clearly conveys how the approach is distinct from -and complementary to - other chemical analytical tools used alongside cryo-ET and cryo-EM, supported by convincing new results highlighting isotope detection. The revised methodological description is also clearer overall, and the annotated photographs of the instrumental setup are especially helpful.

The authors also include yeast lamellae as a eukaryotic proof-of-principle example, demonstrating differential ion distributions in a more complex cytosolic environment. Given that the authors report analyzing ~20 lamellae, but only show two examples, it would be wonderful if they could add a brief summary of what they learned from this dataset; for example, whether any consistent trends emerged across lamellae, and whether particular distribution patterns could be linked to organelles identifiable in the corresponding cryo-ET/cryo-EM data. As one example, the K abundance and distribution appear different between Figure 3f and Supplementary Figure 7i; it would be helpful to know whether the authors think this reflects biological variability, different membrane compartments, differences in lamella/sample preparation, or other experimental factors. If drawing broader conclusions from the current yeast dataset is challenging without additional targeted experiments, perhaps the authors could comment instead.

Overall, the presented cryo-FIB-SIMS method and the correlative cryo-EM/cryo-ET workflow represent an exciting new spatio-chemical imaging approach and a valuable addition to the correlative cryo-EM and cryo-ET toolkit and I am looking forward to its publication. The remaining point for me is minor and relates only to providing a bit more context on the yeast lamella results, if feasible. The revised manuscript is very well and clearly written.

We thank the reviewer for their comments and can indeed report that we see interesting trends in ion distribution across the yeast cells we analysed. In both biological replicates we observed consistent trends between the cells across all lamellae, especially regarding ionic distributions in the cytosol versus compartments visible in the cryo-EM images that correspond to the nucleus or to vacuoles. Across both replicates, most of the major observable ion species in positive mode (K, Na, amino acid fragments) were localised in the cytosol. However, we observed differences in the Mg signal localised within intracellular compartments, which was much more prominent in the second replicate. Given the limited sizes of the data sets, an in-depth interpretation of these observations is challenging, however, our data indicates that this cryo-EM-FIB-SIMS can indeed be useful for studying ionic content and localisation and related biological questions in targeted experiments in eukaryotic cells.

We have addressed this in the manuscript (line 166-170):
"Ionic species such as K and Na showed strong localization in the cytosol across all imaged cells, whereas Mg appeared mainly within intracellular compartments. While an in-depth interpretation of these observations would require targeted experiments, these results clearly demonstrate the generalizability of the workflow to eukaryotic cells."

Reviewer #3 (Remarks to the Author):

Hannah Ochner and colleagues thoroughly revised their exellently-written manuscript titled

"Sub-cellular chemical mapping using correlated cryogenic electron and mass spectrometry imaging"

on a novel correlative workflow to analyze structure and chemical composition of bacteria at sub-cellular level. In their revised manuscript the authors included the missing references to important publications in the field of correlative microscopy of biological specimens under cryogenic conditions and discussed how their work is complementary to these studies. The authors modified parts of the discussion and pointed out how ToF-SIMS, being the central analytical technique in their workflow, allows for localizing molecules of interest on sub-cellular or, in case of eukaryotic cells, sub-organellar level. In my first review I challenged the authors with the question why they are using a ToF-SIMS instead of an energy-dispersive X-ray spectrometer as their major application was the identification of chemical elements in the sample. They correctly argued that SIMS has the advantage to provide information about molecules ad isotopes and proved it by publishing the list of detected ions in negative extraction mode. As a proof-of-principle an additional experiment with 13C labelled cells was conducted which I

find very convincing.
Overall, I think this is an excellent piece of work that in my opinion is now ready for publication in Nature Methods.

We thank the reviewer for their comments.
